# Automated refinement of macromolecular structures at low resolution using prior information

**DOI:** 10.1107/S2059798316014534

**Published:** 2016-09-30

**Authors:** Oleg Kovalevskiy, Robert A. Nicholls, Garib N. Murshudov

**Affiliations:** a MRC Laboratory of Molecular Biology, Francis Crick Avenue, Cambridge Biomedical Campus, Cambridge CB2 0QH, England

**Keywords:** *REFMAC*5, *ProSMART*, low-resolution refinement, external restraints, *LORESTR*

## Abstract

Since the ratio of the number of observations to adjustable parameters is small at low resolution, it is necessary to use complementary information for the analysis of such data. *ProSMART* is a program that can generate restraints for macromolecules using homologous structures, as well as generic restraints for the stabilization of secondary structures. These restraints are used by *REFMAC*5 to stabilize the refinement of an atomic model. However, the optimal refinement protocol varies from case to case, and it is not always obvious how to select appropriate homologous structure(s), or other sources of prior information, for restraint generation. After running extensive tests on a large data set of low-resolution models, the best-performing refinement protocols and strategies for the selection of homologous structures have been identified. These strategies and protocols have been implemented in the *Low-Resolution Structure Refinement* (*LORESTR*) pipeline. The pipeline performs auto-detection of twinning and selects the optimal scaling method and solvent parameters. *LORESTR* can either use user-supplied homologous structures, or run an automated *BLAST* search and download homologues from the PDB. The pipeline executes multiple model-refinement instances using different parameters in order to find the best protocol. Tests show that the automated pipeline improves *R* factors, geometry and Ramachandran statistics for 94% of the low-resolution cases from the PDB included in the test set.

## Abstract 2

An automated pipeline for low-resolution structure refinement (*LORESTR*) has been developed to assist in the hassle-free refinement of difficult cases. The pipeline automates the selection of high-resolution homologues for external restraint generation and optimizes the parameters for *ProSMART* and *REFMAC*5, improving *R* factors and geometry statistics in 94% of the test cases.

## 1. Introduction   

Poor diffraction quality from macromolecular crystals is a persistent problem: various types of short-range and long-range disorder induced by impurities, imperfect crystal-growth conditions and the natural conformational mobility of macromolecules result in the weakening of high-resolution observations, anisotropic diffraction and other problems (Chernov, 2003 ▸; Shaikevitch & Kam, 1981 ▸; Caylor *et al.*, 1999 ▸). Poor crystal diffraction results in the corresponding data sets having a low information content. Whilst substantial effort has been directed towards improving crystal diffraction quality (Heras & Martin, 2005 ▸), quite often it is technically impossible to achieve high-quality diffraction, especially for large multi-subunit complexes. However, the primary aim of X-ray structure analysis is not to obtain the perfect crystal, but rather to determine atomic models of proteins, nucleic acids or complexes of interest that are of sufficient quality to be of use in addressing questions of biological relevance. Low-resolution crystal data can provide exactly such information. Hence, there is a high demand for the development of techniques to allow crystallographers to build reliable models using incomplete, limited and noisy diffraction data.

An important step in the determination of atomic models of macromolecular structures is model refinement. It has two main purposes: (i) to optimize the agreement between atomic models, experimental data and prior knowledge, and (ii) to produce the ‘best’, most informative, electron-density maps using experimental data as well as the current state of the model. *REFMAC*5 is a refinement program that exploits a Bayesian framework to achieve these objectives. *REFMAC*5 minimizes the target function
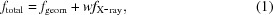
where *f*_geom_ is the contribution of the geometry term (the negative log prior probability distribution, representing our prior chemical and structural knowledge), *f*_X-ray_ is the contribution from the experimentally observed data (the negative log-likelihood function, representing the probability of the data given the current model) and *w* is a weight specifying the relative contributions of these terms (Murshudov *et al.*, 1997 ▸, 2011 ▸). At the end of each refinement session, *REFMAC*5 produces coefficients for weighted 2*F*_o_ − *F*_c_ and *F*_o_ − *F*_c_ maps. Since the phases are calculated from the current state of the model, the quality of these maps depends on the quality of the model. Therefore, reducing overfitting is an important ingredient for improving the signal-to-noise ratio in the calculated maps.

Normally, the geometry term includes restraints on the chemical bond lengths, angles, chiral centres and planarities; the reference values for these restraints are taken from a dictionary that has been calculated using a large number of high-quality experimental structures (Vagin *et al.*, 2004 ▸). In addition, *REFMAC*5 now has tools allowing the introduction of restraints on any desired interatomic distances in the model, the so-called external restraints (Nicholls *et al.*, 2012 ▸). These additional restraints are designed to stabilize refinement in difficult cases where the information content of the diffraction data is low and, consequently, the observation-to-parameter ratio is poor. Introducing external restraints reduces the effective number of adjustable parameters and therefore reduces overfitting of the model into noise. It also changes the landscape of the function to be minimized, thus increasing the radius of convergence of refinement. One of the questions asked when using external reference restraints is: how can we determine which interatomic distances to restrain in difficult refinement cases?

The recently introduced program *ProSMART* (Nicholls *et al.*, 2014 ▸) works in tandem with *REFMAC*5 by supplying additional restraints on interatomic distances using known structural models of homologous proteins, using backbone hydrogen bonds detected in the refined structure or using a library of standard backbone conformations corresponding to secondary-structure elements. For the first case (external restraints based on homologous structures) *ProSMART* performs local structural alignment of the target and reference chains, identifying matching atoms. Then, for every atom in the reference chain that matches an atom in the target chain, the program searches within a particular distance (*e.g.* 4.2 Å) for neighbouring atoms that are not covalently bound. *Pro­SMART* then records the interatomic distances found in the reference structure(s) as the objective values of the restraints, which are subsequently used by *REFMAC*5 during refinement of the target structure. In order to use such external restraints, one or more reference structures that are sufficiently similar to the target must be available. In the case of hydrogen-bond restraints, *ProSMART* detects potential hydrogen bonds between main-chain atoms in the target structure under refinement (no reference structures are needed) and uses a standard hydrogen-bond length (2.8 Å) as the objective value when generating corresponding restraints for use by *REFMAC*5; these restraints help to maintain the structural integrity of the main-chain conformation at low resolution (4–5 Å). In the last case (standard geometry library) *ProSMART* detects α-helical and β-strand-like fragments in the target structure; the distances found in the reference structures, which are taken from a library of typical conformations, are then used as the objective values of the restraints. *ProSMART* has proven to be a useful tool for aiding refinement of difficult cases at low resolution. However, many decisions (selection of homologues for restraint generation, choosing the optimal mode and parameters for both *ProSMART* and *REFMAC*5) are left to the user, and obtaining the best possible results requires substantial manual effort and optimization of parameters through trial and error. In addition to the *REFMAC*5–*ProSMART* tandem from the *CCP*4 suite (Winn *et al.*, 2011 ▸), various other modern macromolecular refinement software tools can utilize additional structural information in different forms, such as secondary-structure restraints, homologous reference structures and homology models. For example, additional structural information can be used by *BUSTER-TNT* (Blanc *et al.*, 2004 ▸, Smart *et al.*, 2012 ▸), *phenix.refine* (Adams *et al.*, 2010 ▸; Headd *et al.*, 2012 ▸), *SHELX* (Sheldrick, 2008 ▸) and *CNS* (Schröder *et al.*, 2010 ▸).

As a general trend, programs for macromolecular crystallography are moving towards full automation, including the handling of difficult cases. Previously, it was believed that only expert crystallographers could successfully deal with the most difficult cases, requiring the use of all of their expertise and practical and theoretical knowledge. However, quite often, human experts follow some sort of algorithm that is applicable to a whole class of analogous cases: they analyse several indicators, assess the case and develop a strategy for dealing with such a scenario. If the algorithm used by an expert can be formalized, then it can potentially be implemented as a computer program that may be used to solve such difficult cases with minimal user intervention. There are a number of examples of successful automation for handling difficult crystallographic cases. For instance, *REFMAC*5 needs only one keyword ‘TWIN’, without any additional parameters, in order to automatically detect the number of twin domains, determine twin operators and estimate twin fractions without any user intervention (Murshudov *et al.*, 2011 ▸). During the last few years, substantial progress has been made towards automation of the whole macromolecular structure-determination process. For instance, a number of automated pipelines for molecular replacement, structure solution and refinement have been reported (Winter *et al.*, 2013 ▸; Long *et al.*, 2008 ▸; Keegan *et al.*, 2011 ▸; Minor *et al.*, 2006 ▸; Wojdyr *et al.*, 2014 ▸; Bibby *et al.*, 2012 ▸). However, they mostly aim at dealing with more or less standard cases. Taking into account the increasing complexity of the biological objects analysed using X-ray crystallography, there is a high demand for the automated handling of nontrivial cases, such as those in which diffraction data with only low resolution or low completeness are available.

*ProSMART* is a tool that has proven to be helpful for refinement in low-resolution cases (Li *et al.*, 2016 ▸; Reich *et al.*, 2014 ▸; Bai *et al.*, 2015 ▸). However, we found that the performance of the generated restraints greatly depends on the selection of homologous structure(s) and the parameters used. This means that, in order to find the optimal protocol, users would typically need to spend a substantial amount of time trying to refine the target structure using various sets of external restraints generated using different homologues and parameters, especially when models for multiple homologues are available. The purpose of the current work is to systematically investigate the factors contributing to the success of refinement by *REFMAC*5 (Murshudov *et al.*, 1997 ▸, 2011 ▸) when using *ProSMART*-generated external restraints, to rationalize the basis of selecting homologous protein models to achieve optimal refinement performance and to implement these findings in a fully automated pipeline that refines difficult low-resolution cases with minimal user intervention.

## 2. Methods   

*REFMAC*5 v.5.8.0107 (Murshudov *et al.*, 1997 ▸, 2011 ▸) and *ProSMART* v.0.843 (Nicholls *et al.*, 2014 ▸) were used in all tests. 16% of the target low-resolution structures in the test set were detected as twinned by *REFMAC*5; these were treated as twinned (by specifying the ‘TWIN’ *REFMAC*5 keyword) in all tests. Details of the exact *REFMAC*5 and *ProSMART* parameters used can be found in Appendices *A* [App appa] and *B* [App appb]. Refinement quality was assessed using *R*_free_ (Brünger, 1992 ▸) and the *MolProbity* score percentile (Chen *et al.*, 2010 ▸). *MolProbity* assessment was performed using the *MolProbity* implementation from the *PHENIX* suite v.1.10 (Adams *et al.*, 2010 ▸).

Some PDB entries include diffraction data to a resolution higher than indicated in the PDB header. For our analysis, we refined against all data available, using the resolution of the diffraction data (not the resolution reported in the PDB header) to assess model quality, noting that the *MolProbity* score percentile depends on resolution.

### 2.1. Construction of the test sets   

Initially, we screened the PDB to identify models of homologous proteins sharing at least 80% sequence identity, resulting in 11245 nonredundant groups (Long *et al.*, 2008 ▸). We then filtered the groups according to resolution, selecting all groups that had at least one structure (comprising a single protein chain) above 2.9 Å resolution and at least one below 3.0 Å resolution. Finally, we excluded groups in which the X-ray data corresponding to a low-resolution model were not available or had *R*_free_ reflections that were either not assigned or assigned incorrectly (leading to *R*_work_ ≃ *R*_free_). As a result, we ended up with 104 cases in which there was a low-resolution structure (3.0–6.7 Å) with a single protein chain and between one and 49 high-resolution homologues (1.3–2.9 Å) (see Supplementary Table S1 for details).

## 3. Optimal refinement protocols for low-resolution cases   

### 3.1. Testing basic refinement protocols   

**Figure 1:**
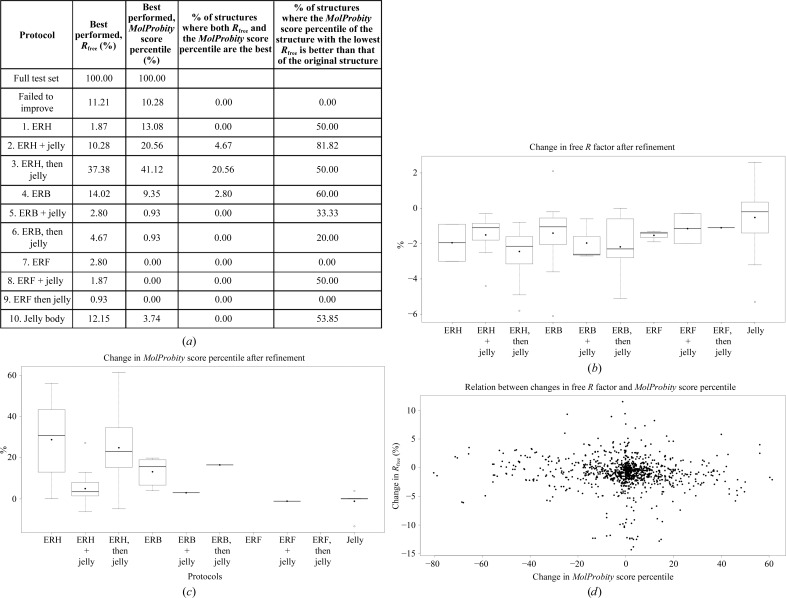
Performance of the tested protocols (*a*), using the same nomenclature as in Table 1 ▸. Where appropriate, external restraints were generated using the one homologue that had the most similar conformation (lowest global r.m.s.d.) to the target structure. (*b*) and (*c*) show box plots representing the distributions of changes in *R*_free_ and the *MolProbity* score percentile, respectively, after refinement using different protocols. All data correspond to the results arising from the protocol that displayed the best performance for the particular structure (lowest *R*_free_ after refinement). In all box plots the mean is shown as a black dot and the median as a thick horizontal line. (*d*) displays a scatter plot showing the relationship between the change in *R*_free_ and the change in *MolProbity* score percentile, for all protocols, for the whole test set.

All test cases were refined using ten protocols, as described in Table 1 ▸. For the protocols that required external restraints from a homologous structure, the single homologue that was found to have the lowest global r.m.s.d. to the target low-resolution structure was used for restraint generation. Using these ten protocols, *R*_free_ was improved for 88.9% of the structures in the test set (see Fig. 1 ▸); the average improvement in *R*_free_ was 2.5%.

The best-performing protocol that showed the maximal improvement in *R*_free_ for the maximal number (37.4%) of structures in the test set was protocol 3: refinement with external restraints from a single homologue followed by a second round of refinement using jelly-body restraints. The average decrease (improvement) in *R*_free_ for this protocol was 3.5%. One can easily imagine how the first refinement run using external restraints brings the structure to a new more realistic conformation that is closer to the conformation of the homologue. The subsequent refinement run using only jelly-body restraints allows the structure to gently relax into the observed data, allowing the atoms to better describe the experimental data, thus resulting in a lower *R*_free_.

The second best-performing protocol, showing the best performance for 14% of the structures in the test set, was protocol 4: refinement with external main-chain hydrogen-bond restraints. In this case, no new information from any external homologues was supplied during refinement. The average improvement in *R*_free_ was 2.6%.

The worst-performing protocols were protocols 7–9, which were based on the library of fragments with idealized geometry. Also, protocol 1 (standard restrained refinement with external restraints from a high-resolution homologue alone) performed quite poorly, showing the best performance in only two cases (1.9%). According to our observations, an additional *REFMAC*5 run using only jelly-body restraints is required in order to allow relaxation of the model, allowing the refinement to find a better local minimum in the new model configuration.

We also assessed the quality of the re-refined models using the *MolProbity* score percentile (Chen *et al.*, 2010 ▸), which was improved for 89.7% of the test cases. The protocol that improved the *MolProbity* percentile score for the maximal number of structures was again achieved with protocol 3 (refinement with external restraints from a single homologue followed by an additional *REFMAC*5 run using jelly-body restraints), in which the average increase in the *MolProbity* score percentile was 25.5%. Again, the worst-performing protocols were protocols based on the library of fragments with idealized geometry (also, in a few outstanding cases these protocols disturbed the geometrical quality of the structures, resulting in a dramatic decrease in the *MolProbity* score percentile by 60–80%; see the left part of Fig. 1 ▸ *d*). Interestingly, for a given structure the protocol that produced the best *R*_free_ value did not necessarily also produce the best *MolProbity* score (see Fig. 1 ▸ *d*). For instance, only 20.6% of the structures in the test set showed both the lowest *R*_free_ value and the highest *MolProbity* score when using protocol 3. However, the majority of the re-refined models showed some improvement in *MolProbity* score over that of the original structure when re-refinement was performed using the protocol that resulted in the lowest *R*_free_. This emphasizes an interesting question: how can the quality of refinement protocols using both the geometric quality of the structure and the correspondence between the model and experimental X-ray diffraction data (indicated by *R* factors) be assessed?

### 3.2. Testing additional refinement options   

There are other parameters that could potentially affect refinement at low resolution. Therefore, we re-executed the refinements with some additional options enabled (one at a time) in order to assess their effects. We compared the minimal *R*_free_ value achieved (using any of the ten protocols) with and without enabling the additional options.

Firstly, we checked the effect of the automatic addition of H atoms in their riding positions. For 93% of the test structures, this option increased the minimal *R*_free_ value using the best-performing protocol (the mean increase was 0.55% and the maximal increase was 1.8%); a minor improvement of minimal *R*_free_ (0.3% on average, 0.7% maximal improvement) was observed for only 7% of the test set. Consequently, we conclude that it is not possible to widely use this option for improving refinement at resolutions below 3.0 Å.

Whilst the current implementation of *REFMAC*5 has an algorithm to automatically determine the weight between the X-ray and geometric components, we tested whether the explicit specification of weights could substantially improve the refinement process (‘WEIGHT MATRIX’ parameter). We re-executed all basic protocols for the whole test set, using *REFMAC*5 ‘WEIGHT MATRIX’ parameters from 0.005 to 0.1 in increments of 0.005. This time, we observed an improvement of the best *R*_free_ values for 20% of the test structures (the mean improvement was 0.26% and the maximal improvement was 0.8%). However, in 80% of the cases explicit specification of the weight term resulted in a mean increase in the minimal *R*_free_ by 0.38% (the maximal observed increase was 1.7%). Another complication we faced was that the best-performing weights appeared to be very different for different protocols and for different test structures, with no apparent correlation with parameters such as resolution. Thus, in order to achieve a minor improvement in *R*_free_, a long-running scan of a wide range of weights would be required (20 additional *REFMAC*5 runs would be required, compared with just two runs at this stage). Owing to this, despite observing that explicit specification of the weight can improve refinement for 20% of cases, we decided to use the auto-weighting in *REFMAC*5, which performed well for the majority of test cases.

### 3.3. Selection of homologous structures for the generation of external restraints   

In our preliminary tests, we observed that the efficiency of the external restraints generated by *ProSMART* depends on the appropriate selection of homologous structures used for restraint generation. Consequently, we further investigated this issue.

#### 3.3.1. Sequence identity of the homologues   

**Figure 2:**
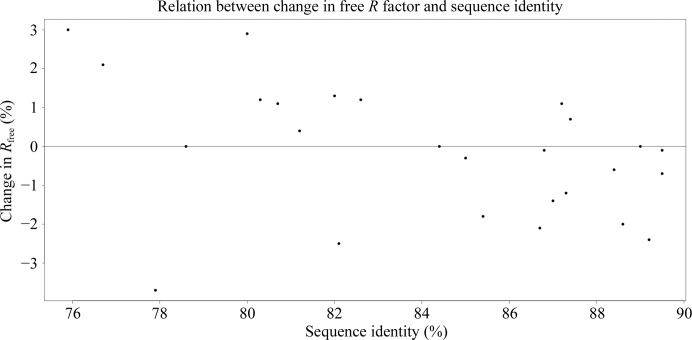
Dependence of the change in free *R* factor on the sequence identity of the high-resolution homologue used for external restraint generation. Note that a difference in *R*_free_ represents a difference between refinement with external restraints using protocol 3 and refinement with jelly-body restraints alone.

Firstly, we asked what the minimal sequence identity between the low-resolution target structure and a reference homologue can be, whilst still being able to benefit from external restraints during refinement. From the test set, we selected the 17 cases where one of the high-resolution homologues had sequence identity in the range 75–90%, and used this single homologue for restraint generation (Supplementary Table S1, subset 2). Refinement protocol 3 was used (external restraints from a homologue followed by a separate jelly-body run) given that it was the most successful protocol identified during previous tests. Unsurprisingly, a clear dependence of refinement success on sequence identity was observed (Fig. 2 ▸). For sequence identities over 85%, external restraints improved refinement in most cases, resulting in *R*_free_ being lower than when re-refining the structure using jelly-body restraints alone (differences in the range 0.2–2.4%). For homologues with sequence identity in the range 75–85%, in most cases refinement resulted in *R*_free_ values higher than after refinement with jelly-body restraints alone but lower than the starting value. In a few cases, an increase in *R*_free_ compared with the starting value was observed, indicating that using relatively distant homologues can have a negative impact on refinement. However, there are exceptions: in one case, using a homologue with 78% sequence identity resulted in a dramatic decrease in *R*_free_ by 4.3%, whilst refinement of the same structure using jelly-body restraints decreased *R*_free_ by only 1.1% (low-resolution structure PDB entry 1jkt, high-resolution homologue PDB entry 2yab). Therefore, in rare cases, even homologues with sequence identity as low as 78% might be helpful. However, for reliable performance, homologues with a sequence identity above 85% are generally required.

#### 3.3.2. Number of homologues for restraint generation   

Next, we explored the possibility of using restraints from several homologues simultaneously. *ProSMART* can generate restraints using any number of homologues. If multiple homologues are available, *ProSMART* will produce several alternative restraints for the same pair of atoms, and during refinement *REFMAC*5 will select the restraint that is the closest to the current interatomic distance in the target structure. For our tests, we limited the test set to the 97 cases (Supplementary Table S1, subset 1) where at least two high-resolution homologues were available for each low-resolution target, and we again used protocol 3: refinement with external restraints followed by a separate jelly-body run.

We found that compared with using the single homologue with the lowest global r.m.s.d., using restraints generated from all available homologues resulted in *R*_free_ being improved in 24% of cases (1.3% on average; the maximal decrease was an impressive 2.8%). However, in 35% of cases *R*_free_ was higher (1.5% on average and maximally increased by 4.9%). We tried to understand the basis for such a difference and found the only obvious difference between these two groups to be in the number of homologues. The group where additional homologues improved refinement had 3.5 homologues on average (with a maximum of eight), whereas in the group were multiple homologues had a negative impact on refinement there were 5.6 homologues on average (with a maximum of 14). Following on from this observation, we hypothesized that generating restraints using fewer homologues performs better.

We selected the subset of 25 structures (Supplementary Table S1, subset 3) for which using multiple homologues showed a better or equal performance compared with using just the one homologue with the minimal global r.m.s.d.. For each of these cases, we used protocol 3 to re-refine the model using restraints generated from each of the available high-resolution homologues, using just one homologue per run. We then ranked the high-resolution homologues according to the resultant *R*_free_ after refinement. A correlation between global r.m.s.d. value and success of refinement was observed; in general, the more structurally similar (measured as a lower global r.m.s.d.) the homologue was to the target structure, the better the external restraints worked (measured as a lower *R*_free_ after refinement). Contrary to our expectations, refinement success showed no clear dependence on average local backbone r.m.s.d. (*i.e.* the ‘Flexible score’ calculated by *Pro­SMART* by superposing nine-residue fragments over the whole chain). However, combining the local and global r.m.s.d. scores (using a simple sum of these two values) turned out to be a better indicator than global r.m.s.d. alone. The sum of local and global r.m.s.d. scores correctly predicted 74% of the best-performing homologues, while global r.m.s.d. alone predicted only 67%. Consequently, we decided to use the sum of local and global r.m.s.d. scores as a measure by which to rank homologues, allowing us to order the homologues according to predicted effectiveness for restraint generation. In fact, in all cases from this subset of 25 structures, the actual best-performing homologue was always found to be in one of the top three places according to our ranking system.

Testing all available single homologues (one per run) identified only four out of the 25 cases where restraints from one of the single homologues outperformed (*R*_free_ lower by 0.1–0.3%) restraints from multiple (all available) homologues; using multiple homologues performed better in 21 out of 25 test cases. Of the 25 cases, 11 had exactly two high-resolution homologues available; the other 14 had three or more.

We then asked the question: what is the minimal number of homologues required to reproduce the low *R*_free_ value observed when using all available homologues? We started to iteratively re-refine the models, adding one more homologue for restraint generation per run, using our ranking system in order to decide which homologues to add. We found that for ten cases out of 25 the addition of a second homologue was sufficient to result in an *R*_free_ equal to or better than the *R*_free_ observed when using all available homologues. For all other cases, addition of the third homologue resulted in an *R*_free_ equal to or better than that observed when using all available homologues. Hence, restraints generated from the top two or three ranked homologues were sufficient to reproduce the best *R*_free_ obtained when using all available homologues for the 25 structures. Interestingly, in five cases using just two or three homologues actually performed better than using all available homologues (three cases with two homologues and two cases with three homologues, with *R*_free_ differences in the range 0.1–0.3%). Also, in five cases restraints generated using just two homologues performed better than when using three homologues.

The above tests were performed only for the cases where the use of multiple homologues showed a better or equal performance relative to using just the one ‘best’ homologue (that with the lowest global r.m.s.d.). However, there were 34 cases in which the use of restraints generated using all available homologues performed worse than using just the one homologue with the lowest global r.m.s.d.. We re-refined these 34 structures (Supplementary Table S1, subset 4) using restraints generated from every single available homologue separately (one homologue per run), as well as combinations of the two and three top homologues, as ranked using our system. We also tried using two additional protocols: external hydrogen-bond restraints and jelly-body restraints, neither of which refer to any external homologous structures. In 20 cases, the best performance (*i.e.* the lowest *R*_free_) was indeed realised when using restraints from just the one single homologue with a minimal global r.m.s.d. score; this result could not be improved by any of the other protocols trialled. For five cases, the best performance was obtained when using the top two ranked homologues and for five cases when using the top three ranked homologues.

Surprisingly, we found six cases for which the best performance was realised when using the homologue with one of the largest global r.m.s.d. scores (three cases with the largest global r.m.s.d, one case with the second largest and two cases with the third largest global r.m.s.d.); *i.e.* the structure with a global conformation most different from that of the current state of the target model. This was unexpected, as it contradicts our previous assertion that homologues corresponding to the lowest global r.m.s.d. score generally perform best. We hypothesize that this apparent contrary behaviour is owing to the presence of different classes of problems: the optimal way in which to refine a low-resolution model is dependent on the current state and quality of the model and the stage in the refinement process. Specifically, if the current quality of the low-resolution model is good (*i.e.* the model reasonably closely resembles the crystal contents) then the best performance may be realised when using external restraints from a high-resolution homologue with low global r.m.s.d. to the low-resolution model. However, if the current quality of the low-resolution model is poor, and the model does not resemble the actual atomic positions in the crystal sufficiently well, then homologues exhibiting more different conformations (thus having large global r.m.s.d. scores) may better resemble the actual crystal contents. Consequently, external restraints from such homologues may be more effective. Furthermore, the fact that restraints from such homologues would encourage the model to adopt a different conformation may have a positive effect by allowing the model to escape local minima during refinement. We speculate that one cause of this kind of scenario, in which the current state of the low-resolution model is particularly poor, could be the result of poor selection of the structure used for molecular replacement. Consequently, we recommend trying all (structurally nonredundant) available models for molecular replacement and comparing the results as a matter of course when only low-resolution data are available.

Out of the remaining four structures, two performed best when using hydrogen-bond restraints and two when using jelly-body restraints alone. Most likely this means that all available homologues for these test cases have a global and/or local structure that is too distant from that of the protein chain in the target crystal for the corresponding external restraints to have a positive effect on refinement. It is clear that the use of external restraints pushes the target structure towards the conformation of the reference structure(s), introducing bias towards our ‘prior knowledge’. Such bias could have a positive or negative effect, depending on how structurally similar the reference model is to the true structure in the target crystal, as well as on the quality of the current model. If all available reference structures have different local conformations to the target model, it could be more beneficial to refine the structure using only jelly-body regularization, or alternatively to use hydrogen-bond restraints to help to maintain the backbone conformation. Indeed, the success of refinement with external restraints depends on the availability of high-quality homologous structural models with local conformations similar to that in the target crystal.

### 3.4. Assessing refinement quality using *R*_free_ and *MolProbity* score   

Ideally, refinement efficiency should be assessed using both *R*_free_ (which reflects the correspondence between the model and the observed diffraction data; Brünger, 1992 ▸) and the stereochemical quality of the resulting model (Chen *et al.*, 2010 ▸), especially when comparing several different refinement protocols using the same starting model and data. As mentioned above, we observed that in roughly a quarter of the test cases the protocol that returned the refined model with the lowest *R*_free_ also delivered the model with the best stereochemical quality (see Figs. 1*a* and 1*d* ▸). In many cases, the protocol that produced a model with substantially better geometry also resulted in one of the best *R*_free_ scores, with only minor differences (in the range 0.1–0.3%) separating the top scores. In other cases, protocols that produced substantially better *R*_free_ values would produce models with only slightly worse geometric quality than the best protocol (see Supplementary Fig. 1 ▸).

We decided to develop a single synthetic measure (*Q*-score) for the purpose of ranking refinements according to their overall success, using a combination of statistics representing the quality of model geometry (based on *MolProbity* score percentiles) and the goodness-of-fit of the model to the data (represented by *R*_free_). After manually examining various test cases and trying several different empirical equations, we found the following measure to produce the most reasonable ranking of the refinement protocols,
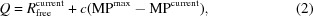
where *R*_free_^current^ and MP^current^ are the *R*_free_ and *MolProbity* score percentile values after refinement using the current protocol, the weight *c* is defined as the ratio between the range of *R*_free_ values and the range of *MolProbity* score percentiles,
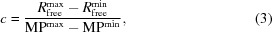
where *R* ^min^_free_ and *R* ^max^_free_ are the minimum and maximum *R*_free_ values observed over all protocols (for this case), and MP^min^ and MP^max^ are the minimum and maximum *MolProbity* score percentiles observed over all protocols.

In the degenerate case in which all refinement protocols result in identical *MolProbity* percentile values (so that MP^min^ and MP^max^ are equal), the weighting term *c* is set to zero.

This *Q*-score essentially inflates *R*_free_ for protocols that do not show the best geometric quality, with the increase being directly proportional to the relative difference between the geometric quality of the best and current models. The protocol with the lowest *Q*-score is ranked the best.

## 4. Automated pipeline for refinement at low resolution   

Using a test set of 104 cases, we attempted to identify the best parameters and combinations of protocols for improving refinement at low resolution using *REFMAC*5 with the assistance of external restraints generated by *ProSMART*. Every crystallographic case is different. For instance, structural models of high-resolution close homologues may be available for some target proteins, but not for others. This makes it almost impossible to design one universal refinement protocol that would be optimal for all possible cases. Also, we could not find a strong correlation between refinement protocol performance and any of the obvious main parameters, such as the resolution of the data, the geometric quality of the model, the values of the *R* factors *etc*. Consequently, we concluded that the most appropriate strategy, which strikes a good balance between reliability and efficiency, is to first identify the minimal set of top-performing protocols and then try using all of these protocols in order to find the one which performs best. Ideally, the minimal set of protocols should be such that one of the protocols produces optimal refinement results in all cases. We have attempted to identify such a minimal set of refinement protocols, and have implemented them in a refinement pipeline.

**Figure 3:**
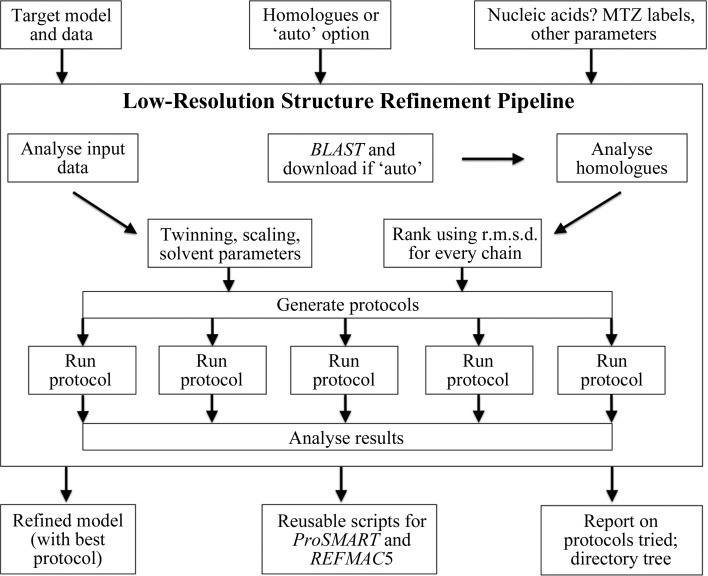
Flow diagram of the *Low-Resolution Structure Refinement* (*LORESTR*) pipeline.

The *Low-Resolution Structure Refinement* (*LORESTR*) pipeline (available in *CCP*4 v.7.0; Collaborative Computational Project, 1994 ▸; Winn *et al.*, 2011 ▸) was designed to be a fully automated and easy-to-use tool (Fig. 3 ▸). The minimal input required by the pipeline is a PDB file containing the current model (the target structure) and an MTZ file containing the corresponding diffraction data. In automatic mode, it extracts the sequences of all chains present in the PDB file and runs a *BLAST* search over the whole Protein Data Bank (internet connection required; Berman *et al.*, 2000 ▸; Altschul *et al.*, 1990 ▸). It then downloads all homologues that share at least 75% sequence identity and cover at least 75% of the protein chain (these default values arose from the tests described in §[Sec sec3.3.1]3.3.1). *LORESTR* specifies that *ProSMART* hydrogen-bond restraints should be used for any chains for which no close homologues are found. Users can also manually supply any number of homologous structures (PDB files). This is useful, for instance, in cases in which the PDB files are private and/or not yet released in the PDB.

After downloading homologues, the pipeline analyses the input data in order to determine the set of most appropriate refinement parameters. It checks whether the data are derived from a twinned crystal, in which case automated handling of twinning in *REFMAC*5 is enabled. The pipeline also tries standard {resistant scaling based on log[cosh(*F*_o_ − *F*_c_)]} and least-squares scaling options, selecting the one that performs better (gives the lower *R*_free_). After that, the pipeline uses *ProSMART* to analyse and match all input chains from all supplied homologues; chains are ranked using the sum of average local and global r.m.s.d. scores. Homologous chains with a sequence identity below 75% or with less than 75% coverage of the target chain are rejected.

The pipeline then generates a number of refinement protocols, depending on the number of available homologous chains. Provided that sufficient chains are available for external restraint generation, *LORESTR* generates and executes refinement protocols using the one, two and three best-ranked homologues, as well as the one, two and three homologues with the largest global r.m.s.d. scores, one protocol using restraints from all available homologues, and finally two protocols that do not require any information from the homologous structures: hydrogen-bond restraints and jelly-body restraints. However, users may specify the desired number of homologues, should they wish for more than (or less than) three homologues to be used. If no homologues are supplied and no homologues are found during the *BLAST* search, the pipeline will just test the two protocols that do not require the availability of external homologues, *i.e.* hydrogen-bond restraints and jelly-body restraints. For all protocols for which external homologues are available, the pipeline runs one round of *REFMAC*5 refinement using the external restraints before then executing a second round of refinement using only jelly-body restraints in order to allow the structure to relax into its new conformation (as this approach proved to be optimal in the vast majority of our test cases).

*LORESTR* generates command files for *ProSMART* and *REFMAC*5 for each protocol, before executing the appropriate programs. The pipeline supports multitasking and can run several jobs in parallel, should the user wish. After running all jobs, *LORESTR* selects the best-performing protocol according to the *Q*-score (see §[Sec sec3.4]3.4), or just simply *R*_free_ if *MolProbity* is not available from a local *PHENIX* (Adams *et al.*, 2010 ▸) installation (in the current implementation, *LORESTR* looks for the presence of *phenix.molprobity* in the current user’s path; we will switch to *MolProbity* distributed with *CCP*4 once it has been released). The refined PDB and MTZ files corresponding to the best protocol are returned, along with a *LORESTR* protocol file. *LORESTR* protocol files can be supplied as an input in subsequent executions of the pipeline, in which case only the supplied protocol will be executed. This allows quick refinement of the target structure using only this protocol, *e.g.* after further model rebuilding and refinement. Alternatively, experienced users may access the *ProSMART* and *REFMAC*5 command scripts directly.

The pipeline also recognizes several optional parameters. For instance, users can specify nonstandard MTZ file column labels, supply TLS definitions (Winn *et al.*, 2001 ▸), change the number of protein chains or homologues used for external restraint generation and specify the number of CPUs used when running refinement jobs in parallel. Another useful option is for automated refinement directly after molecular replacement. In this case, before running standard refinement protocols, the pipeline runs 100–200 cycles (depending on the starting *R* factors) of refinement using jelly-body restraints in order to relax the structure into its new position.

**Figure 4:**
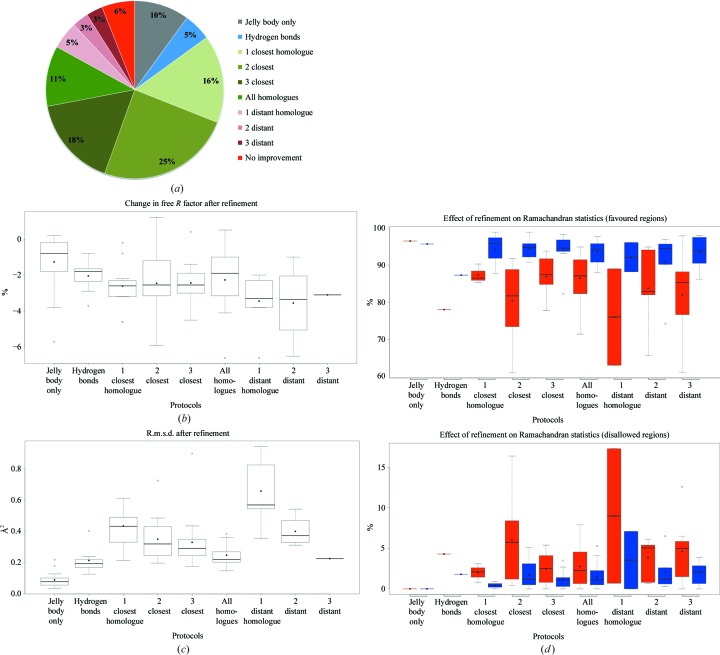
Efficiency of the protocols implemented in the pipeline. (*a*) Pie chart illustrating how often each protocol performed best according to the *Q*-score. (*b*) Box plots illustrating the change in *R*_free_ after refinement for each protocol. Data shown correspond to the structures for which the given protocol performed best. Analogous box plots show the change in global r.m.s.d. between atomic positions (*c*) and Ramachandran statistics (*d*) for the models before and after refinement. Values before refinement are shown in red and those after refinement in blue.

We tested the performance of *LORESTR* using our test set of 104 structures, finding that the pipeline improved *R*_free_ in 94% of cases (see Fig. 4 ▸). Protocols using *ProSMART* external restraints produced the best-refined models in 84% of test cases: 79% using external restraints from homologous structure(s) and the remaining 5% using hydrogen-bond restraints. In 10% of cases, the use of jelly-body restraints alone proved optimal; most likely this reflects a substantial difference in the conformation of the available high-resolution homologue(s) compared with that of the protein in the target crystal.

As seen in Figs. 4*b* and 4*c* ▸, minimal improvement of both *R*_free_ and r.m.s.d. (between the models before and after refinement) was observed for protocols using jelly-body restraints only (average of 1.4% and 0.148 Å, respectively) and hydrogen-bond restraints (average of 2.0% and 0.165 Å, respectively). These results are not surprising, as the other protocols that make use of high-resolution models of homologous structures inject more new structural information and thus have a greater potential to improve the models.

Quite consistently, the maximal average r.m.s.d. between the models before and after refinement (0.64 Å) was observed for the protocol with external restraints from the one most conformationally different homologue (Fig. 4 ▸ *c*). However, protocols using the two and three most distant homologues resulted in slightly smaller structural changes (global r.m.s.d. difference) that were more akin to that observed when using the closest homologue. The maximal average improvement of *R*_free_ (3.4%) was observed for the protocol using the two most distant homologues; the change in *R*_free_ for the protocols with most distant homologues was generally larger compared with the protocols using the closest homologues. This suggests that in these cases the original models before refinement were in conformations that were quite different from that of the real protein structure in the target crystals. Furthermore, the homologues exhibiting the highest global r.m.s.d.s to the current state of the models were in fact closer in structure to the contents of the real target crystals. Quite logically, using external restraints in these cases induces substantial conformational changes in the refined models (as indicated by high r.m.s.d. values before and after refinement), resulting in impressive drops in *R*_free_. However, only 11% of the test structures could be improved using these protocols; these cases question the quality of the original models deposited in the Protein Data Bank.

For most of the test cases (57%), protocols using a few close homologues showed the best performance, resulting in reasonable reductions in *R*_free_ (2.4–2.6%) and exhibiting small conformational changes (global r.m.s.d. of 0.3–0.4 Å) during refinement.

For several cases, manual inspection of the electron-density maps after refinement showed improved connectivity and the occasional appearance of new features in the density. To assess the effect of external restraints on the resulting models, we examined the overall real-space electron-density map (2*mF*_o_ − *DF*_c_) correlation and Ramachandran statistics before and after refinement.

Overall electron-density map correlation showed only a minor average change after refinement, varying substantially from structure to structure; increased correlation after refinement was observed in some cases and decreased correlation in others. Manual examination of several of these structures suggests that model rebuilding, guided by the improved electron-density maps, would be required after refinement with external restraints; the use of external restraints often reveals new features in the density, providing opportunities for further model improvement.

A strikingly different picture was observed for the Ramachandran statistics (Fig. 4 ▸ *d*). Whilst jelly-body restraints resulted in negligible changes in the Ramachandran plot, all of the protocols based on external restraints displayed a dramatic improvement in the statistics: the number of residues in favourable regions increased by 5–17% and the number of residues in disallowed regions decreased by 1–3%. Using external restraints derived from high-resolution homologues pushes the target structure towards the conformations of those homologues. Since such homologues typically have substantially better geometric, stereochemical and other properties, the external restraints cause improvement of these properties in the low-resolution target models. This is a perfect illustration of the benefits of using homologue-based external restraints during low-resolution model refinement.

### 4.1. Examples of usage   

As illustrative examples, we have picked five test cases from the top 20 low-resolution structures that showed the most substantial decrease in *R*_free_ using the *LORESTR* pipeline in auto mode; details of these cases are presented in Table 2 ▸. The original quality of these deposited structures, as judged by *MolProbity*, was found to be below average (all *MolProbity* score and clashscore percentiles were well below 50). Note that these five structures, which are those that were most improved by *LORESTR*, are all structures with poor original geometric quality. This reflects the very simple idea that it is difficult to further improve the quality of well refined structures, whilst poor-quality models can be substantially improved using automated approaches. Indeed, for these structures, in addition to improved *R* factors, we see dramatic improvement of the geometric quality (*MolProbity* and clashscores improved by around 40–60 percentiles). We observed an amazing improvement of the Ramachandran statistics, despite the fact that they are not explicitly used as refinement targets; the number of Ramachandran outliers reduced by roughly fivefold in many cases. The overall r.m.s.d. for these structures before and after refinement varied from 0.4 to 0.7 Å.

During manual inspection of the electron-density maps for these cases, we found that the visual quality of the maps did not change substantially. Sometimes we could see minor improvements, such as better connectivity of the blobs representing bulky side chains and the main chain. One possible explanation for the electron-density maps appearing very similar, despite *R*_free_ decreasing by 3–4%, is the reduction in model bias when *REFMAC*5 calculates map coefficients; owing to excessive model bias, the original maps are misleading, being of poorer quality than they appear.

During the course of our investigation, we found an interesting case: a model of the structure of the insulin receptor tyrosine kinase in complex with a ligand (PDB entry 2z8c), which was solved using data extending to 3.25 Å resolution. The deposited model has reasonably poor geometric quality, having a clashscore of 28.2, a *MolProbity* score percentile of 28.2%, 5.0% Ramachandran outliers and only 78.2% of residues in favoured Ramachandran regions (Table 3 ▸). According to the PDB header, PDB entry 2z8c was solved by molecular replacement using another model (PDB entry 1ir3) determined at 1.9 Å resolution; this high-resolution structure has good geometric quality (clashscore 2.72, 98.0% of residues in favoured Ramachandran regions and 0.3% outliers). Evidently, the rebuilding and refinement process affected the geometric quality of 2z8c: superposition of 2z8c and 1ir3 clearly shows two regions in which the models have diverged owing to substantial rebuilding and retracing of the main chain. Multiple Ramachandran outliers can be found in these regions. We tried using *LORESTR* in auto mode to see whether the model could be automatically improved. Indeed, *LORESTR* was able to improve 2z8c, resulting in a model with lower *R* factors and better geometry (see Table 3 ▸). The best-performing protocol used external restraints from all available homologues (five homologues were automatically found: PDB entries 5e1s, 4ibm, 1ir3, 1gag and 1irk). Again, this demonstrates the substantial positive utility of introducing *ProSMART* external restraints during refinement.

We also tried to completely resolve the structure from scratch. We used *MOLREP* (Vagin & Teplyakov, 2010 ▸) to obtain the initial model and phases, performing molecular replacement using PDB entry 1ir3 as the search model (*i.e.* the same search model as was used for the original deposited structure 2z8c) and the deposited X-ray data for PDB entry 2z8c. The ligand was manually added to the model by copying it from the original 2z8c structure. Without further intervention/rebuilding, we then immediately ran the *LORESTR* pipeline, resulting in a dramatic improvement of both *R* factors and model geometry (Table 3 ▸). This case demonstrates how external restraints can improve models even in cases where the structure has been substantially (negatively) affected by model alteration and rebuilding, However, in such cases the atomic positions may be too far from their ideal positions (too far out of the radius of convergence) for the external restraints to be able to correct the model during refinement. Indeed, there is no substitute for starting from a better model; for the best result one may need to return back to the molecular-replacement stage and start again (using the *LORESTR* pipeline).

**Figure 5:**
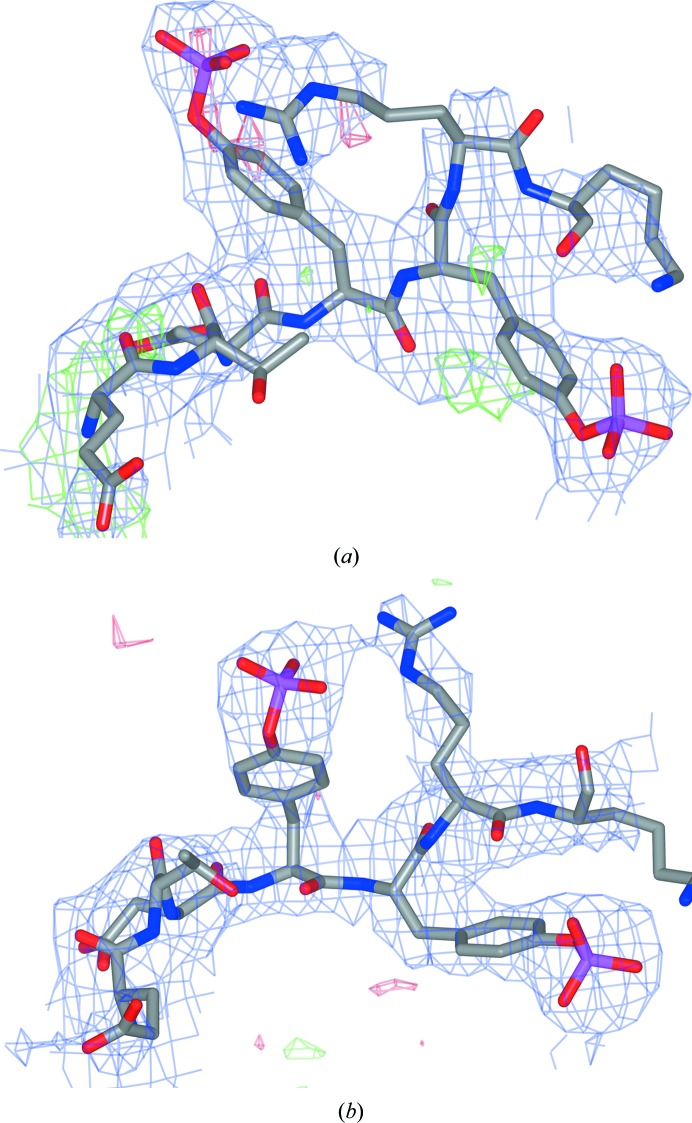
Comparison of 2*mF*_o_ − *DF*_c_ electron-density maps (contoured at 1σ) and difference maps (3σ) around residues 1159–1165 for the original model from PDB entry 2z8c (*a*) and for the model resolved using PDB entry 1ir3 as the search model and refined with *LORESTR* (*b*).

After resolving 2z8c by molecular replacement, the best-performing refinement protocol became jelly-body only; external restraints could not improve the model any further, as the starting model was already of very good quality. Fig. 5 ▸(*a*) displays electron-density maps for the region around residues 1159–1165 in the original deposited model of PDB entry 2z8c, and Fig. 5 ▸(*b*) shows the corresponding maps for the model resulting from *LORESTR* after resolving 2z8c using PDB entry 1ir3 as the molecular-replacement search model. The original model has four Ramachandran outliers in the displayed region, and difference-map peaks suggest an imperfect fit of the model to the density. Residues in the corresponding region after resolving and refinement with *LORESTR* exhibit no Ramachandran outliers, and it is evident that the model better fits the density. Note also that the model adopts a substantially different conformation in this whole region.

In general, if stuck with refinement at low resolution, we recommend returning to the molecular-replacement step and trying molecular replacement using models of different homologous structures with substantially different conformations (reasonably high r.m.s.d.), as there is chance that other homologues may model the X-ray diffraction data better and ultimately lead to a better refined model. Executing *LORESTR* directly after each molecular-replacement trial (with a different homologue as the search model) provides a quick and easy way to determine which starting models and phases might lead to optimal refinement success.

## 5. Discussion   

We have tested various refinement strategies as well as different *REFMAC*5 and *ProSMART* parameters on a test set of more than 100 structures. We found that in cases where high-resolution homologues are available the best strategy is to first execute a *REFMAC*5 refinement run using external restraints generated by *ProSMART*, followed by a second round of refinement using only jelly-body restraints. The availability and the selection of appropriate high-resolution homologues is important for successful refinement using external restraints. Such homologues should have a local conformation sufficiently close to that of the true structure in the target low-resolution crystal, which is typically the case for proteins sharing at least 75% sequence identity. In cases where no homologues are available for a particular protein chain, external restraints representing backbone hydrogen bonds can improve refinement.

In most cases in which multiple models of high-resolution homologues are available, using external restraints generated from just the one, two or three homologues with the closest global conformation (lowest global r.m.s.d. to the low-resolution model under refinement) produces better results than using all available homologues. Interestingly, sometimes refinement with external restraints generated from homologues with a substantially different conformation (highest global r.m.s.d.) from that of the target structure can result in a dramatic decrease in *R*_free_, substantial structural rearrangement, better geometry and overall improvement of the target model. The fact that the most conformationally different homologues can be the best choice of homologues to use for external restraint generation implies that in such cases the structures of those homologues may better represent the low-resolution crystal contents than the original models deposited in the PDB. This could be the result of suboptimal selection of homologues for initial molecular replacement. Therefore, we recommend trying all available structures (with sufficiently different conformations) for molecular replacement, subsequently executing the *LORESTR* pipeline for each solution, and comparing the results.

The best-performing protocols have been implemented in *LORESTR*: an automated pipeline for structure refinement at low resolution, distributed as part of the *CCP*4 suite (Winn *et al.*, 2011 ▸). The pipeline facilitates the fully automated selection of optimal external restraints from *ProSMART* for structure refinement by *REFMAC*5. It can automatically run a *BLAST* search to identify homologues, and download the corresponding models from the PDB. It automatically detects twinning, and finds the optimal scaling method and parameters for solvent modelling. The pipeline runs a number of refinement protocols in order to find the best protocol for each particular case. In our tests, *LORESTR* was able to produce substantially better quality models in the vast majority of cases, improving both *R* factors and model geometry for 94% of test cases. The dramatic improvement in *R* factors and the geometric quality of low-resolution models observed when using the fully automated mode of the pipeline demonstrates its potential use for researchers working with low-resolution cases, especially during the initial stages of refinement, or when unable to further progress with refinement.

Future development of the pipeline may include multi-crystal refinement: treatment of the special case where several low-resolution X-ray diffraction data sets and models are available for a particular protein. In this case, we can attempt to co-refine all structures simultaneously, executing multiple concurrent *REFMAC*5 refinements, generating external restraints for each model using all others and iterating until convergence. This procedure would allow information transfer between the structures, which we anticipate could potentially improve the refinement and thus the quality of the resulting models.

## Supplementary Material

Supporting Information.. DOI: 10.1107/S2059798316014534/rr5131sup1.pdf

## Figures and Tables

**Table 1:** The numeration and abbreviations used in the text for different combinations of external restraint and refinement protocols, involving external restraints to homologous structures (ERH), hydrogen bonds (ERB) and restraints based on library of fragments (ERF)

External restraint type	Homologous structure†	Hydrogen bonds	Fragment library	
Standard refinement with external restraints	1. ERH	4. ERB	7. ERF	10. Single round of refinement using jelly-body restraints
Jelly-body and external restraints in the same run	2. ERH + jelly	5. ERB + jelly	8. ERF + jelly	
Run of standard refinement with external restraints followed by a separate jelly-body run‡	3. ERH, then jelly	6. ERB, then jelly	9. ERF, then jelly	

† External restraints were generated using the single homologue that had the most similar conformation (lowest global r.m.s.d.) to the target structure.

‡ Two *REFMAC*5 runs in total; the output from the first run was used as the input for the second.

**Table:** 

		*R*_work_ (%)			*R*_free_ (%)			Difference between *R*_work_ and *R*_free_ (%)		
PDB code	Protocol	Before	After	Change	Before	After	Change	Before	After	R.m.s.d. after refinement (Å)
1dcm	External restraints from all homologues	27.5	24.3	−3.2	34.1	29.8	−4.3	6.6	5.5	0.388
1jkt	External restraints from the two closest homologues	24.3	18.8	−5.5	28.4	22.9	−5.5	4.1	4.1	0.684
1u9o	External restraints from the two closest homologues	21.1	18.4	−2.7	26.1	21.7	−4.4	5.0	3.3	0.406
2bvg	External restraints from the two most different homologues	24.1	20.3	−3.8	27.4	23.5	−3.9	3.3	3.2	0.433
2waf	External restraints from all homologues	28.5	23.0	−5.5	33.0	26.2	−6.8	4.5	3.2	0.438

**Table:** 

		Ramachandran favoured (%)			Ramachandran outliers (%)			*MolProbity* score percentile		
PDB code	Protocol	Before	After	Change	Before	After	Change	Before	After	Change
1dcm	External restraints from all homologues	84.5	93.3	8.8	5.0	2.9	−2.1	12.2	52.3	40.1
1jkt	External restraints from the two closest homologues	61.1	91.6	30.5	16.2	2.9	−13.3	4.6	62.9	58.3
1u9o	External restraints from the two closest homologues	73.4	97.6	24.2	5.7	0.8	−4.9	31.7	92.4	60.7
2bvg	External restraints from the two most different homologues	82.0	96.8	14.8	4.9	0.4	−4.5	37.4	89.1	51.7
2waf	External restraints from all homologues	83.6	95.6	12.0	5.2	0.65	−4.55	14.1	77.4	63.3

**Table 3:** A comparison of refinement and geometry statistics is provided for the original model (PDB entry 2z8c) and for the model resolved using PDB entry 1ir3 as the search model and refined with *LORESTR* (Fig. 5 ▸)

Structure	*R*_work_ (%)	*R*_free_ (%)	Difference between *R*_free_ and *R*_work_ (%)	Ramachandran favoured (%)	Ramachandran outliers (%)	Clashscore	Clashscore percentile	*MolProbity* score	*MolProbity* score percentile
2z8c	21.7	29.2	7.5	78.2	5.0	28.2	33.0	3.41	28.2
After *LORESTR* in auto mode	23.2	25.2	2.0	90.3	4.0	10.5	68.0	2.78	55.1
Resolved with 1ir3, then *LORESTR*	18.6	23.4	4.8	96.0	1.3	5.1	89.0	1.88	90.7

## Appendix A *ProSMART* parameters

The *LORESTR* pipeline runs *ProSMART* for restraint generation with the following parameters.

For homologue-based restraints: prosmart -p1 target.pdb -c1 TargetChain -p2 homologue1.pdb homologue2.pdb -c2 Chain1 Chain2 -restrain_all -side -sigmatype 0.

For hydrogen bond-based restraints: prosmart -p1 target.pdb -c1 TargetChain -bond -sigmatype 0.

## Appendix B *REFMAC*5 parameters

Typical *REFMAC*5 parameters (if twinning is detected then the TWIN keyword is added).

(i) First run with external restraints:

MAKE -
HYDROGEN NO -
NEWLIGAND CONTINUE
NCYC 40
EXTERNAL WEIGHT SCALE 10
EXTERNAL WEIGHT GMWT 0.02
EXTERNAL DMAX 4.2
@restraintFile.txt
MONI DIST 1000000
END

(ii) Following jelly-body run:

MAKE -
HYDROGEN NO -
NEWLIGAND CONTINUE
NCYC 20
RIDG DIST SIGM 0.01
MONI DIST 1000000
END
