# Supplementary material for: Automated refinement of macromolecular structures at low resolution using prior information
Source: Acta Crystallogr D Struct Biol. 2016 Sep 30;72(Pt 10):1149–61. doi: 10.1107/S2059798316014534 (PMC5053141; doi:10.1107/S2059798316014534)
Supplement: Supplementary file 1 [file d-72-01149-sup1.pdf]

STRUCTURAL BIOLOGY

Volume 72 (2016)

Supporting information for article:

Automated refinement of macromolecular structures at low resolution using prior information

Oleg Kovalevskiy, Robert A. Nicholls and Garib N. Murshudov

Figure S1 Supporting information Scatterplots show performance of the LORESTR Q-score ranking on several random test cases; the numeric observation labels indicate the Q-score rank for a particular protocol that resulted in the corresponding R_{free} value and MolProbity percentile score.

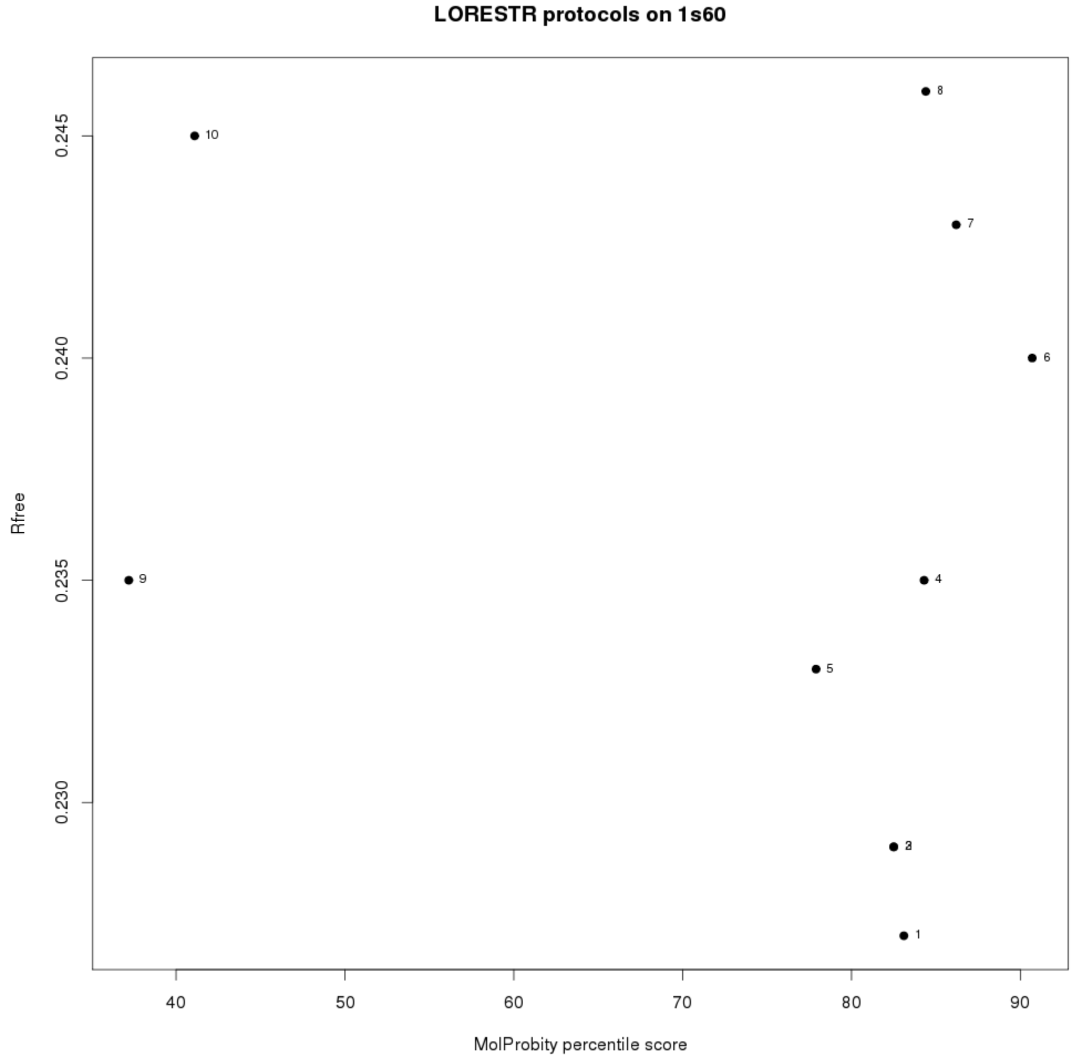

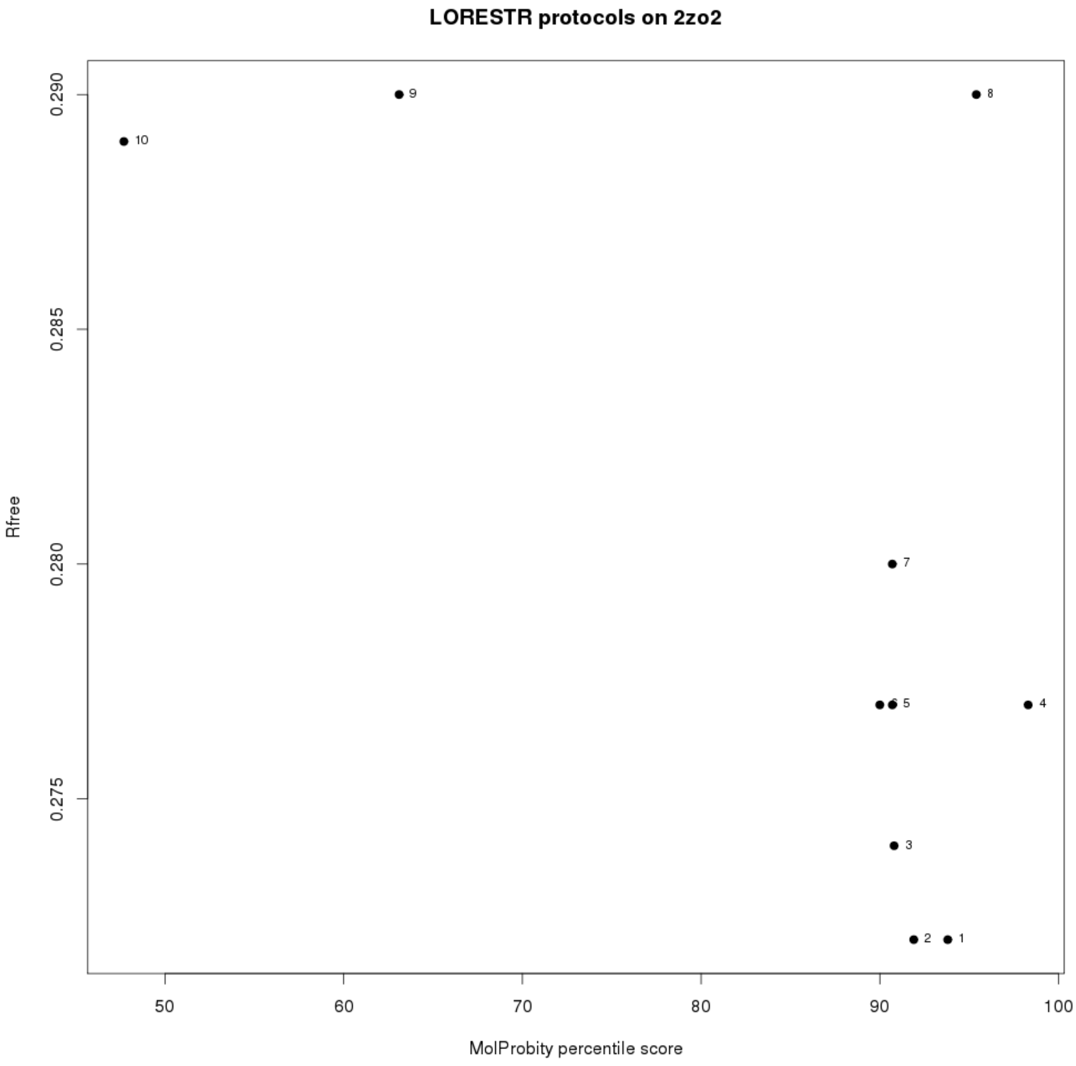
LORESTR protocols on 2zo2
Rfree
0.290
0.285
0.280
0.275
MolProbity percentile score
50
60
70
80
90
100
1
2
3
4
5
6
7
8
9
10

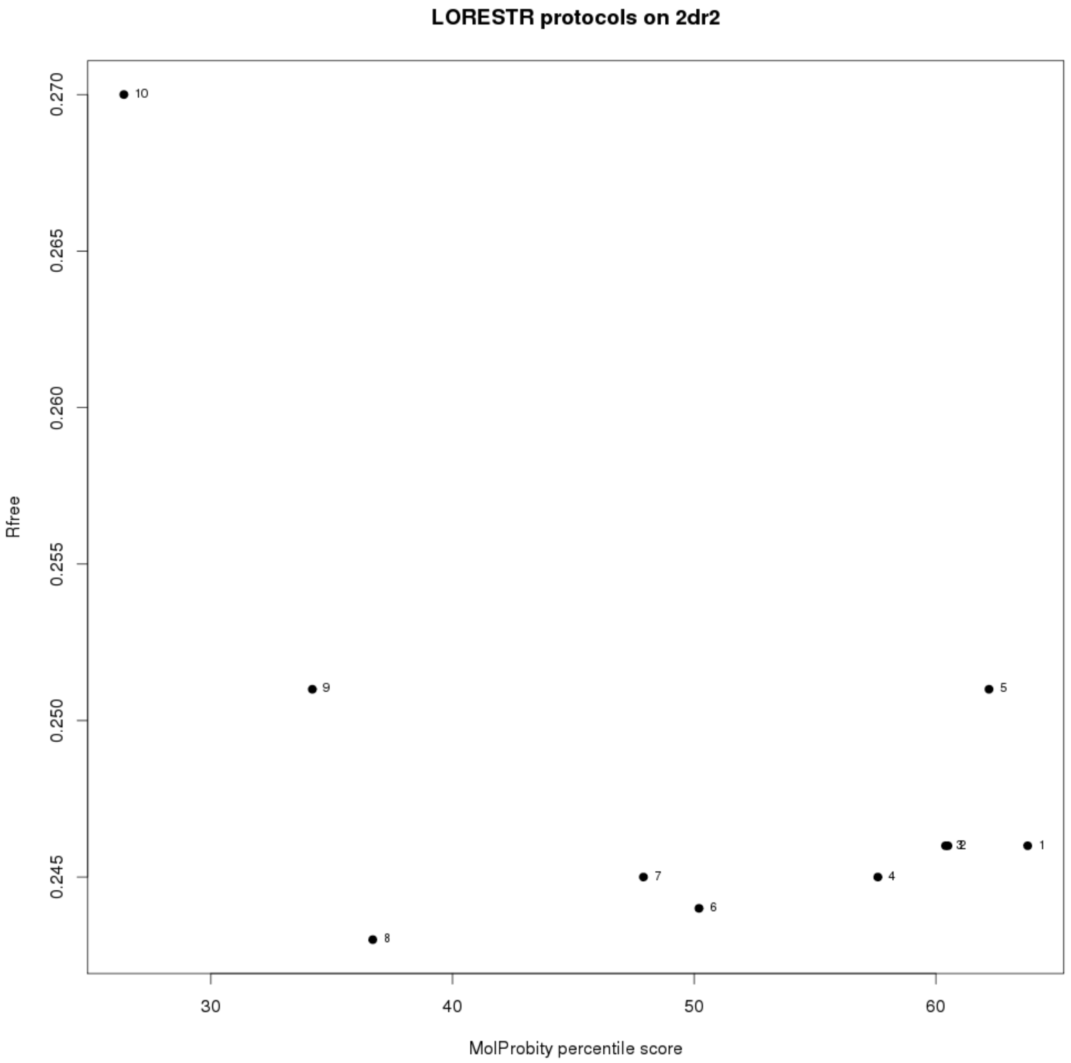
LORESTR protocols on 2dr2
Rfree
0.270
0.265
0.260
0.255
0.250
0.245
30
40
50
60
MolProbity percentile score
10
9
8
7
6
4
3
2
5
1

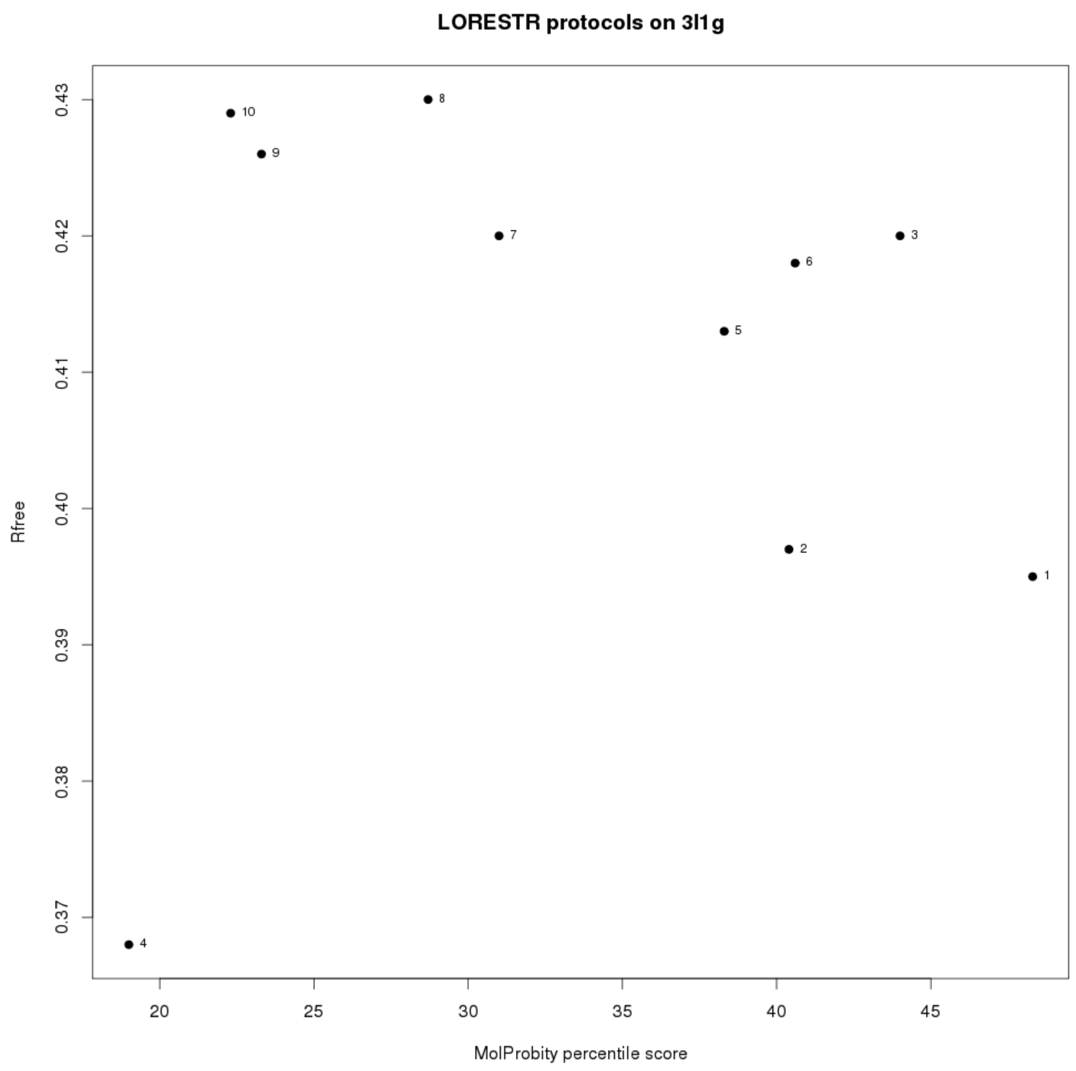
LORESTR protocols on 3l1g
Rfree
MolProbity percentile score
0.43
0.42
0.41
0.40
0.39
0.38
0.37
20
25
30
35
40
45
10
9
8
7
6
5
3
2
1
4

Table S1 Details of the test sets

Subset 1: more than two homologues, used for refinement with multiple homologues

Subset 2: have homologues with 75%-90% sequence identity

Subset 3: cases where multiple homologues performed better or equal to a single homologue

Subset 4: cases where multiple homologues performed worse than a single homologue

PDB code	Reported resolution	Resolution of the data	Subset 1	Subset 2	Subset 3	Subset 4	Number of homologues	High-resolution homologues (PDB code, resolution, sequence identity, RMSD)
2rgr	3.0	3.0	X				3	3l4j (2.5 A, 99.7%, 2.79 A), 1bjt (2.5 A, 100.0%, 15.90 A), 1bgw (2.7 A, 100.0%, 15.20 A),
2is2	3.0	3.0	X		X		3	3lfu (1.8 A, 99.8%, 14.50 A), 2is6 (2.2 A, 100.0%, 3.01 A), 2is1 (2.9 A, 100.0%, 1.22 A),
1ob5	3.1	3.0	X				7	2c78 (1.4 A, 98.0%, 4.41 A), 2c77 (1.6 A, 97.8%, 4.01 A), 1exm (1.7 A, 97.5%, 2.34 A), 1ha3 (2.0 A, 97.4%, 1.35 A), 1b23 (2.6 A, 100.0%, 2.58 A), 1ttt (2.7 A, 100.0%, 2.54 A), 1tui (2.7 A, 100.0%, 11.10 A),
2c9m	3.0	3.0	X			X	11	3ar7 (2.1 A, 100.0%, 15.40 A), 3n5k (2.2 A, 100.0%, 15.50 A), 1wpg (2.3 A, 100.0%, 15.30 A), 2agv (2.4 A, 100.0%, 15.40 A), 2zbf (2.4 A, 100.0%, 14.30 A), 2zbd (2.4 A, 100.0%, 13.90 A), 1su4 (2.4 A, 100.0%, 4.61 A), 3fpb (2.5 A, 100.0%, 15.40 A), 3b9b (2.6 A, 100.0%, 14.10 A), 1vfp (2.9 A, 100.0%, 14.00 A), 2eat (2.9 A, 100.0%, 15.50 A),
3b9r	3.0	3.0	X			X	11	3ar7 (2.1 A, 100.0%, 4.41 A), 3n5k (2.2 A, 100.0%, 1.43 A), 1wpg (2.3 A, 100.0%, 1.46 A), 2agv (2.4 A, 100.0%, 4.46 A), 2zbf (2.4 A, 100.0%, 2.60 A), 2zbd (2.4 A, 100.0%, 11.60 A), 1su4 (2.4 A, 100.0%, 13.40 A), 3fpb (2.5 A, 100.0%, 1.55 A), 3b9b (2.6 A, 100.0%, 3.81 A), 1vfp (2.9 A, 100.0%, 11.80 A), 2eat (2.9 A, 100.0%, 4.39 A),

2pkg	3.3	3.3	X				4	1b3u (2.3 A, 100.0%, 6.24 A), 2ie4 (2.6 A, 100.0%, 5.59 A), 3fga (2.7 A, 99.8%, 2.83 A), 3dw8 (2.9 A, 100.0%, 2.05 A),
3s4u	3.3	3.3	X		X		3	3p7i (1.7 A, 99.7%, 7.71 A), 3quj (2.2 A, 97.6%, 7.99 A), 3qk6 (2.4 A, 97.7%, 7.90 A),
1lwt	3.2	3.2	X				3	1dfa (2.0 A, 98.0%, 3.80 A), 1jva (2.1 A, 97.5%, 1.31 A), 1ef0 (2.1 A, 97.8%, 2.44 A),
1lws	3.5	3.5	X				3	1dfa (2.0 A, 98.0%, 3.80 A), 1jva (2.1 A, 97.5%, 1.30 A), 1ef0 (2.1 A, 97.8%, 2.77 A),
1u88	3.5	3.5	X			X	6	1dug (1.8 A, 99.5%, 0.69 A), 3crt (1.9 A, 99.0%, 0.76 A), 1m9a (2.1 A, 99.5%, 0.67 A), 4ecb (2.2 A, 99.5%, 1.47 A), 1gta (2.4 A, 99.5%, 0.68 A), 1b8x (2.7 A, 99.5%, 0.83 A),
3ks2	3.3	3.3	X				2	3gz1 (2.1 A, 100.0%, 7.46 A), 3gyz (2.1 A, 100.0%, 5.61 A),
1a7b	3.1	3.1	X			X	2	1cdc (2.0 A, 100.0%, 7.32 A), 1a64 (2.0 A, 100.0%, 0.76 A),
2dut	3.0	3.0	X				3	2h4p (1.7 A, 100.0%, 6.97 A), 2h4q (2.1 A, 97.6%, 4.49 A), 2h4r (2.7 A, 100.0%, 1.17 A),
1l5t	3.0	3.0	X				6	1h45 (1.9 A, 98.7%, 6.26 A), 1h44 (2.0 A, 99.4%, 6.01 A), 1dsn (2.0 A, 99.0%, 6.77 A), 1hse (2.2 A, 99.1%, 6.07 A), 1vfe (2.3 A, 99.4%, 6.31 A), 1vfd (2.5 A, 99.4%, 6.30 A),
1jkt	3.5	3.5	X	X		X	13	3bhy (1.2 A, 82.1%, 1.26 A), 2w4j (1.3 A, 100.0%, 1.44 A), 2a2a (1.5 A, 77.9%, 1.72 A), 3bqr (1.8 A, 81.7%, 6.39 A), 2xuu (1.8 A, 99.6%, 1.55 A), 2yab (1.9 A, 78.2%, 1.60 A), 2w4k (1.9 A, 100.0%, 1.56 A), 2j90 (2.0 A, 81.1%, 6.08 A), 2xzs (2.0 A, 100.0%, 1.68 A), 2x0g (2.2 A, 100.0%, 1.52 A), 1wvw (2.4 A, 100.0%, 1.49 A), 2y4p (2.6 A, 100.0%, 5.49 A), 1zws (2.9 A, 77.9%, 1.65 A),
2atq	3.2	3.2	X		X		5	3qex (1.7 A, 99.6%, 6.21 A), 3l8b (2.1 A, 100.0%, 3.27 A), 1ih7 (2.2 A, 99.8%, 2.73 A),

								2dtu (2.4 A, 99.9%, 3.13 A), 1clq (2.7 A, 99.8%, 2.32 A),
3vqk	4.5	4.5	X				4	3aab (1.9 A, 98.1%, 4.28 A), 3vql (1.9 A, 100.0%, 1.69 A), 3aac (2.4 A, 98.1%, 7.16 A), 3vqm (2.5 A, 100.0%, 1.64 A),
3dpn	3.3	3.2	X			X	3	3dor (2.2 A, 97.1%, 6.04 A), 3dpm (2.4 A, 96.7%, 6.42 A), 3dja (2.9 A, 95.8%, 6.40 A),
3e3j	6.7	6.7	X			X	4	1msw (2.1 A, 99.9%, 16.00 A), 1qln (2.4 A, 99.9%, 6.34 A), 1aro (2.8 A, 99.6%, 6.16 A), 1h38 (2.9 A, 99.9%, 16.60 A),
2waf	3.3	3.3	X			X	2	2wad (2.2 A, 91.1%, 6.22 A), 2wae (2.3 A, 90.9%, 6.21 A),
2hzv	3.1	3.1	X				2	2hza (2.1 A, 100.0%, 5.64 A), 1q5v (2.3 A, 98.2%, 4.70 A),
3lco	3.4	3.4	X			X	3	2i1m (1.8 A, 95.8%, 1.52 A), 3lcd (2.5 A, 99.3%, 3.65 A), 2ogv (2.7 A, 98.6%, 1.81 A),
3idq	3.7	3.7	X			X	4	2woj (2.0 A, 100.0%, 2.62 A), 3zs9 (2.1 A, 100.0%, 2.54 A), 3h84 (2.3 A, 100.0%, 2.43 A), 3a36 (2.8 A, 99.6%, 2.37 A),
1gub	3.1	3.0	X		X		2	1gud (1.7 A, 100.0%, 1.05 A), 1rpj (1.8 A, 100.0%, 3.95 A),
1t38	3.2	3.2	X	X			3	3l00 (1.7 A, 86.8%, 1.12 A), 1qnt (1.9 A, 99.3%, 0.76 A), 3kzy (1.9 A, 86.8%, 1.11 A),
3l8j	3.0	3.0	X		X		2	3ajm (2.3 A, 100.0%, 3.77 A), 3l8i (2.5 A, 100.0%, 1.74 A),
1w8b	3.0	2.8	X				6	2bz6 (1.6 A, 100.0%, 0.98 A), 2c4f (1.7 A, 100.0%, 1.02 A), 2flb (1.9 A, 100.0%, 1.25 A), 1jbu (2.0 A, 100.0%, 3.94 A), 1fak (2.1 A, 100.0%, 1.13 A), 1qfk (2.8 A, 100.0%, 1.19 A),
3p39	3.1	3.1	X	X		X	14	3rvc (1.8 A, 92.4%, 0.78 A), 2rhk (1.9 A, 87.4%, 1.63 A), 3o9r (2.0 A, 92.4%, 0.73 A), 3m5r (2.0 A, 82.6%, 2.30 A), 2gx9 (2.1 A, 85.4%, 1.68 A), 3ee9 (2.1 A, 85.2%, 2.36 A), 3o9t (2.2 A, 82.8%, 1.72 A), 3kwg (2.2 A, 86.1%, 2.39 A), 3l4q (2.3 A, 90.0%, 3.18 A),

								3p31 (2.5 A, 99.2%, 1.81 A), 3o9s (2.5 A, 90.9%, 1.81 A), 3o9q (2.5 A, 91.7%, 0.86 A), 3ee8 (2.6 A, 85.2%, 2.42 A), 3p38 (2.8 A, 100.0%, 0.32 A),
3o9u	3.2	3.2	X	X			14	3rvc (1.8 A, 99.1%, 0.72 A), 2rhk (1.9 A, 89.7%, 1.70 A), 3o9r (2.0 A, 99.1%, 0.82 A), 3m5r (2.0 A, 87.1%, 1.81 A), 2gx9 (2.1 A, 95.7%, 0.72 A), 3ee9 (2.1 A, 89.7%, 1.73 A), 3o9t (2.2 A, 100.0%, 1.52 A), 3kwg (2.2 A, 88.8%, 1.78 A), 3l4q (2.3 A, 100.0%, 1.89 A), 3p31 (2.5 A, 92.2%, 1.85 A), 3o9s (2.5 A, 100.0%, 1.37 A), 3o9q (2.5 A, 99.1%, 0.69 A), 3ee8 (2.6 A, 89.7%, 1.88 A), 3p38 (2.8 A, 91.4%, 0.92 A),
1ob2	3.4	3.4	X			X	10	1efc (2.0 A, 100.0%, 11.20 A), 2hcj (2.1 A, 99.4%, 11.00 A), 3u6b (2.1 A, 99.7%, 10.80 A), 2bvn (2.3 A, 100.0%, 3.41 A), 1d8t (2.4 A, 99.7%, 11.20 A), 3u6k (2.5 A, 99.7%, 11.60 A), 1efu (2.5 A, 100.0%, 11.80 A), 1dg1 (2.5 A, 100.0%, 11.10 A), 3u2q (2.7 A, 99.7%, 11.80 A), 2hdn (2.8 A, 99.7%, 11.20 A),
2fx3	3.4	3.4	X				10	1efc (2.0 A, 99.7%, 1.21 A), 2hcj (2.1 A, 99.7%, 1.30 A), 3u6b (2.1 A, 100.0%, 1.30 A), 2bvn (2.3 A, 99.7%, 11.80 A), 1d8t (2.4 A, 100.0%, 1.25 A), 3u6k (2.5 A, 100.0%, 1.25 A), 1efu (2.5 A, 99.7%, 2.68 A), 1dg1 (2.5 A, 99.7%, 1.46 A), 3u2q (2.7 A, 100.0%, 1.09 A), 2hdn (2.8 A, 100.0%, 1.38 A),
2cxf	3.1	3.1					1	2dwk (2.0 A, 100.0%, 3.36 A),
2cxl	3.2	3.2					1	2dwk (2.0 A, 100.0%, 2.91 A),
1juj	3.0	3.0	X				12	3gh0 (1.6 A, 99.6%, 3.02 A), 4e5o (1.7 A, 93.4%, 2.85 A), 4eb4 (1.7 A, 93.4%, 0.52 A), 1hvy (1.9 A, 99.3%, 0.58 A), 3ihi (1.9 A, 93.2%, 0.71 A), 1hzw (2.0 A, 98.9%, 1.01 A), 3n5e (2.3 A, 98.5%, 2.41 A), 2rd8 (2.5 A,

								99.3%, 1.27 A), 2tsr (2.6 A, 92.9%, 0.78 A), 3h9k (2.6 A, 99.3%, 1.10 A), 2rda (2.7 A, 99.3%, 1.45 A), 3gg5 (2.8 A, 99.6%, 1.05 A),
1fvf	3.2	3.2	X				2	1epu (2.4 A, 96.5%, 3.05 A), 1fvh (2.8 A, 100.0%, 2.32 A),
1rfq	3.0	3.0	X	X			49	2fxu (1.4 A, 99.7%, 0.97 A), 1wua (1.4 A, 99.7%, 0.88 A), 2v52 (1.4 A, 100.0%, 1.20 A), 1qz5 (1.4 A, 99.7%, 1.31 A), 3mn5 (1.5 A, 99.1%, 1.88 A), 2pbd (1.5 A, 99.7%, 1.34 A), 1j6z (1.5 A, 99.7%, 1.53 A), 3cip (1.6 A, 91.4%, 1.08 A), 2asm (1.6 A, 100.0%, 0.99 A), 1d4x (1.8 A, 93.9%, 1.11 A), 3sjh (1.8 A, 100.0%, 0.84 A), 2pav (1.8 A, 99.7%, 1.14 A), 2hf3 (1.8 A, 93.3%, 1.12 A), 2a40 (1.8 A, 99.7%, 1.19 A), 2a42 (1.9 A, 99.7%, 1.09 A), 2hmp (1.9 A, 99.7%, 1.34 A), 2gwj (1.9 A, 99.7%, 1.23 A), 1yag (1.9 A, 87.3%, 1.08 A), 1t44 (2.0 A, 100.0%, 1.22 A), 2gwk (2.0 A, 99.7%, 1.12 A), 3mn7 (2.0 A, 93.3%, 1.07 A), 3mn6 (2.0 A, 93.3%, 1.12 A), 3m6g (2.0 A, 100.0%, 0.95 A), 1esv (2.0 A, 99.7%, 1.06 A), 1c0g (2.0 A, 90.9%, 1.04 A), 3u8x (2.0 A, 100.0%, 1.02 A), 1yxq (2.0 A, 99.7%, 0.94 A), 3u9z (2.1 A, 100.0%, 1.38 A), 1kxp (2.1 A, 100.0%, 0.90 A), 1mdu (2.2 A, 99.7%, 1.18 A), 3ub5 (2.2 A, 94.2%, 1.60 A), 3chw (2.3 A, 91.4%, 1.07 A), 1rdw (2.3 A, 100.0%, 0.99 A), 2vyp (2.4 A, 100.0%, 1.11 A), 2v51 (2.4 A, 100.0%, 1.00 A), 1ma9 (2.4 A, 99.7%, 1.03 A), 4efh (2.5 A, 92.8%, 1.08 A), 2a5x (2.5 A, 100.0%, 0.85 A), 3el2 (2.5 A, 93.3%, 1.05 A), 2q97 (2.5 A, 99.7%, 1.04 A), 2q36 (2.5 A, 100.0%, 0.93 A), 2d1k (2.5 A, 99.7%, 1.21 A), 3daw (2.5 A, 100.0%, 1.63 A), 1p8z (2.6 A, 100.0%, 0.94 A), 2oan (2.6 A, 94.1%, 1.06 A),

								2q1n (2.7 A, 100.0%, 0.99 A), 3ue5 (2.8 A, 100.0%, 1.10 A), 1atn (2.8 A, 99.7%, 1.31 A), 3buz (2.8 A, 100.0%, 1.01 A),
3n6s	3.1	3.1	X		X		2	3mva (2.2 A, 100.0%, 1.78 A), 3n7q (2.4 A, 100.0%, 3.05 A),
3oz0	3.0	3.0	X			X	6	2awh (2.0 A, 99.6%, 1.61 A), 3gz9 (2.0 A, 99.2%, 2.59 A), 2b50 (2.0 A, 99.6%, 2.34 A), 3sp9 (2.3 A, 100.0%, 2.82 A), 2xyj (2.3 A, 100.0%, 2.86 A), 2q5g (2.7 A, 100.0%, 1.74 A),
3prj	3.1	3.1	X		X		7	2q3e (2.0 A, 100.0%, 0.86 A), 2qg4 (2.1 A, 100.0%, 2.19 A), 3tf5 (2.3 A, 100.0%, 2.67 A), 3khu (2.3 A, 99.8%, 1.82 A), 3itk (2.4 A, 99.8%, 2.38 A), 3ptz (2.5 A, 100.0%, 0.31 A), 3tdk (2.8 A, 100.0%, 0.35 A),
2qnc	3.1	3.1	X		X		3	1e7l (1.3 A, 100.0%, 1.62 A), 1en7 (2.4 A, 99.4%, 2.23 A), 1e7d (2.8 A, 99.4%, 1.24 A),
2qrn	3.4	3.2	X		X		8	1p5z (1.6 A, 100.0%, 0.67 A), 2no7 (1.7 A, 98.7%, 0.78 A), 2zi6 (1.8 A, 99.1%, 1.22 A), 2zia (1.8 A, 98.6%, 0.71 A), 3qeo (1.9 A, 97.8%, 1.73 A), 2zi7 (2.0 A, 98.6%, 0.85 A), 3mjr (2.1 A, 98.7%, 1.56 A), 3ipy (2.5 A, 100.0%, 1.65 A),
4dt0	3.6	3.6	X				2	2dm9 (1.9 A, 100.0%, 2.87 A), 2dma (2.0 A, 96.6%, 2.95 A),
1s60	3.0	3.0	X				2	2vbq (2.0 A, 100.0%, 0.60 A), 1s5k (2.4 A, 100.0%, 2.50 A),
2dpu	3.1	3.1	X			X	3	1bm9 (2.0 A, 99.1%, 2.14 A), 2efw (2.5 A, 100.0%, 1.33 A), 1f4k (2.5 A, 100.0%, 0.74 A),
3l1g	3.3	3.3	X			X	3	2y1y (2.0 A, 98.7%, 1.49 A), 2y1z (2.5 A, 97.6%, 2.49 A), 2wj7 (2.6 A, 100.0%, 1.62 A),
3id2	3.1	3.1	X			X	3	2zpm (1.0 A, 91.9%, 1.17 A), 3id4 (1.6 A, 97.8%, 1.81 A), 3id3 (2.0 A, 98.9%, 2.18 A),
2r8a	3.0	3.0	X			X	6	3pgu (1.7 A, 99.2%, 0.82 A), 3pgs (1.9 A, 99.2%, 0.91 A), 2r88 (2.6 A, 98.6%, 0.59 A),

								1t16 (2.6 A, 99.2%, 0.71 A), 1t1l (2.8 A, 99.2%, 0.90 A), 2r4p (2.9 A, 99.2%, 0.69 A),
2r4n	3.2	3.1	X			X	6	3pgu (1.7 A, 99.2%, 1.94 A), 3pgs (1.9 A, 99.3%, 0.83 A), 2r88 (2.6 A, 98.9%, 0.97 A), 1t16 (2.6 A, 99.5%, 0.59 A), 1t1l (2.8 A, 99.5%, 0.93 A), 2r4p (2.9 A, 99.5%, 0.57 A),
2c86	3.0	3.0	X				2	2gec (1.3 A, 92.5%, 1.91 A), 2bxx (1.9 A, 100.0%, 1.19 A),
3km2	3.1	3.1	X		X		4	3pu7 (1.8 A, 100.0%, 0.36 A), 3hog (1.9 A, 100.0%, 2.28 A), 3km1 (2.0 A, 100.0%, 0.74 A), 1srd (2.0 A, 90.2%, 0.68 A),
3hl9	3.4	3.4	X				2	3hld (2.0 A, 98.5%, 2.06 A), 3hlb (2.5 A, 100.0%, 0.47 A),
3cl9	3.3	3.3	X			X	4	3irm (2.1 A, 99.4%, 1.71 A), 3inv (2.4 A, 99.4%, 1.81 A), 2h2q (2.4 A, 100.0%, 1.50 A), 3kjs (2.5 A, 100.0%, 0.95 A),
1i0e	3.5	3.5	X	X			6	1qh4 (1.4 A, 81.6%, 1.41 A), 1u6r (1.6 A, 96.2%, 1.38 A), 3drb (2.0 A, 80.3%, 1.45 A), 1vrp (2.1 A, 83.9%, 1.44 A), 1g0w (2.3 A, 80.8%, 1.45 A), 2crk (2.4 A, 96.4%, 0.75 A),
4a5t	3.5	3.5					1	4dur (2.5 A, 100.0%, 1.93 A),
3auw	3.6	3.6	X			X	2	3agw (2.2 A, 100.0%, 1.16 A), 2e4f (2.3 A, 100.0%, 1.31 A),
2akq	3.0	3.0	X	X			8	1beb (1.8 A, 99.4%, 0.57 A), 3uev (1.9 A, 98.7%, 1.45 A), 1uz2 (1.9 A, 99.4%, 1.88 A), 3kza (2.0 A, 84.4%, 0.91 A), 1yup (2.1 A, 94.9%, 0.65 A), 3ph5 (2.4 A, 98.7%, 0.58 A), 2q39 (2.5 A, 100.0%, 1.30 A), 2r56 (2.8 A, 98.7%, 1.27 A),
2bxs	3.1	3.1					1	2z5y (2.2 A, 99.8%, 1.22 A),
2zgk	3.0	3.0	X	X		X	12	2zgr (1.9 A, 99.4%, 0.94 A), 2zgl (1.9 A, 100.0%, 0.92 A), 2zgm (1.9 A, 100.0%, 0.90 A), 1ww7 (1.9 A, 88.6%, 1.59 A), 2zgq (1.9 A, 99.4%, 0.96 A), 3afk (1.9 A, 100.0%, 0.95 A), 3m3c (2.0 A, 100.0%, 0.93 A), 3m3o (2.1 A,

								99.4%, 0.90 A), 3m3e (2.1 A, 99.4%, 0.94 A), 3m3q (2.2 A, 100.0%, 0.93 A), 1ww6 (2.2 A, 88.6%, 0.69 A), 2zgu (2.4 A, 99.4%, 1.34 A),
1xcp	3.2	3.2	X		X		8	2afh (2.1 A, 99.7%, 0.98 A), 1fp6 (2.1 A, 99.7%, 1.16 A), 1g20 (2.2 A, 96.5%, 1.74 A), 2nip (2.2 A, 99.7%, 1.04 A), 1g1m (2.2 A, 99.7%, 1.03 A), 2afk (2.3 A, 99.6%, 1.73 A), 1m34 (2.3 A, 99.6%, 1.76 A), 1rw4 (2.5 A, 98.9%, 1.65 A),
1kog	3.5	3.5	X				3	1evl (1.6 A, 100.0%, 0.57 A), 1fyf (1.6 A, 100.0%, 0.56 A), 1evk (2.0 A, 100.0%, 1.65 A),
1u6b	3.1	3.1	X			X	9	1nu4 (1.8 A, 100.0%, 1.64 A), 1urn (1.9 A, 100.0%, 0.88 A), 1m5o (2.2 A, 100.0%, 0.65 A), 3egz (2.2 A, 96.7%, 0.69 A), 1sj3 (2.2 A, 100.0%, 1.23 A), 3mxh (2.3 A, 100.0%, 0.93 A), 1oia (2.4 A, 97.8%, 1.30 A), 2nz4 (2.5 A, 100.0%, 0.66 A), 3cul (2.8 A, 95.7%, 0.74 A),
3iwn	3.2	3.2	X			X	9	1nu4 (1.8 A, 100.0%, 1.44 A), 1urn (1.9 A, 100.0%, 0.64 A), 1m5o (2.2 A, 100.0%, 0.65 A), 3egz (2.2 A, 96.7%, 0.62 A), 1sj3 (2.2 A, 100.0%, 0.59 A), 3mxh (2.3 A, 100.0%, 0.90 A), 1oia (2.4 A, 97.7%, 1.10 A), 2nz4 (2.5 A, 100.0%, 0.57 A), 3cul (2.8 A, 96.7%, 0.68 A),
3p49	3.5	3.5	X				9	1nu4 (1.8 A, 100.0%, 1.85 A), 1urn (1.9 A, 100.0%, 0.88 A), 1m5o (2.2 A, 100.0%, 0.88 A), 3egz (2.2 A, 96.7%, 0.83 A), 1sj3 (2.2 A, 100.0%, 0.89 A), 3mxh (2.3 A, 100.0%, 0.76 A), 1oia (2.4 A, 97.7%, 1.23 A), 2nz4 (2.5 A, 100.0%, 0.87 A), 3cul (2.8 A, 95.7%, 0.91 A),
2dr2	3.0	3.0	X				6	1r6u (2.0 A, 96.8%, 0.99 A), 1r6t (2.1 A, 96.5%, 0.95 A), 2qui (2.4 A, 100.0%, 0.85 A), 1o5t (2.5 A, 100.0%, 1.34 A), 2quk (2.8 A, 100.0%, 1.18 A), 2azx (2.8 A, 96.8%, 0.82 A),

1dcm	3.0	2.6	X			X	2	1dbw (1.6 A, 100.0%, 1.35 A), 1d5w (2.3 A, 99.2%, 1.72 A),
2zni	3.1	3.1	X		X		2	3dsq (2.1 A, 100.0%, 1.50 A), 2znj (2.5 A, 100.0%, 1.59 A),
3fbr	3.5	3.5	X		X		4	3dnu (1.5 A, 97.8%, 1.13 A), 3dnt (1.7 A, 97.6%, 0.80 A), 2wiu (2.4 A, 99.8%, 1.55 A), 3dnv (2.7 A, 99.5%, 1.10 A),
2zys	3.1	3.1	X		X		2	2zyr (1.8 A, 100.0%, 0.63 A), 2zyh (1.8 A, 99.8%, 1.51 A),
1gc4	3.3	3.3	X				4	1b5p (1.8 A, 99.5%, 1.20 A), 5bj3 (2.2 A, 100.0%, 0.71 A), 1gck (2.5 A, 99.5%, 1.16 A), 1bkg (2.6 A, 99.0%, 0.58 A),
1gc3	3.3	3.3	X		X		4	1b5p (1.8 A, 99.5%, 1.02 A), 5bj3 (2.2 A, 100.0%, 0.62 A), 1gck (2.5 A, 99.5%, 1.01 A), 1bkg (2.6 A, 99.0%, 0.69 A),
2zo2	3.1	3.1	X	X		X	11	3fde (1.4 A, 100.0%, 1.44 A), 2zkd (1.6 A, 98.9%, 1.23 A), 3bi7 (1.7 A, 86.5%, 0.78 A), 2zkg (1.8 A, 98.9%, 0.62 A), 2pb7 (1.9 A, 87.0%, 1.05 A), 2zo1 (2.0 A, 100.0%, 1.26 A), 3f8j (2.0 A, 99.0%, 1.24 A), 2zo0 (2.2 A, 100.0%, 1.32 A), 3clz (2.2 A, 87.7%, 1.36 A), 3f8i (2.3 A, 100.0%, 1.19 A), 2zkf (2.5 A, 98.9%, 1.23 A),
4dx0	3.4	3.4	X	X		X	6	2br9 (1.8 A, 72.6%, 1.36 A), 1o9d (2.3 A, 87.2%, 1.02 A), 3axy (2.4 A, 89.5%, 1.34 A), 3e6y (2.5 A, 87.2%, 1.11 A), 3m50 (2.6 A, 89.0%, 0.56 A), 2o98 (2.7 A, 87.3%, 1.06 A),
2x0n	3.2	3.2	X	X			4	3ids (1.8 A, 90.2%, 1.37 A), 1i32 (2.6 A, 80.7%, 1.22 A), 1a7k (2.8 A, 80.7%, 1.23 A), 1gyp (2.8 A, 80.7%, 1.24 A),
1fb5	3.5	3.5	X			X	2	1oth (1.9 A, 98.1%, 1.36 A), 1fvo (2.6 A, 98.1%, 1.25 A),
2xrg	3.2	3.2	X		X		2	3nkq (1.7 A, 98.3%, 1.05 A), 2xr9 (2.0 A, 99.9%, 1.18 A),

1de9	3.0	3.0	X			X	7	2o3h (1.9 A, 99.3%, 0.65 A), 1hd7 (1.9 A, 99.6%, 0.57 A), 3u8u (2.1 A, 99.6%, 0.98 A), 1bix (2.2 A, 99.6%, 0.51 A), 1e9n (2.2 A, 99.6%, 0.73 A), 1dew (2.6 A, 100.0%, 0.66 A), 2isi (2.8 A, 99.6%, 0.85 A),
3uvf	3.0	3.0	X	X		X	2	2qoj (2.4 A, 84.6%, 1.03 A), 3eh8 (2.7 A, 85.4%, 0.75 A),
2fpf	3.0	3.0	X		X		2	2fpe (1.8 A, 96.7%, 0.56 A), 2fpd (2.0 A, 96.7%, 0.50 A),
1u9o	3.3	3.3	X			X	2	3q0w (1.6 A, 100.0%, 1.01 A), 3q0v (1.9 A, 100.0%, 0.83 A),
1xqg	3.1	3.1					1	1wz9 (2.1 A, 97.2%, 1.01 A),
2xz0	3.0	3.0	X	X			3	2uw1 (1.9 A, 82.0%, 0.64 A), 1oq4 (2.4 A, 100.0%, 0.89 A), 1oq9 (2.4 A, 100.0%, 0.74 A),
1nph	3.0	3.0					1	2fh1 (1.6 A, 94.2%, 0.90 A),
2wrf	3.1	3.1	X				3	2wr1 (2.1 A, 93.6%, 1.04 A), 2wr7 (2.5 A, 94.7%, 0.89 A), 2wr4 (2.5 A, 93.0%, 0.93 A),
2nre	4.0	3.5					1	1dj0 (1.5 A, 100.0%, 1.11 A),
1uaz	3.4	3.4	X	X	X		3	2ei4 (2.1 A, 85.0%, 0.70 A), 2z55 (2.5 A, 85.0%, 0.74 A), 1vgo (2.5 A, 86.1%, 0.72 A),
3i78	3.0	3.0	X	X		X	5	3beu (1.1 A, 98.7%, 0.75 A), 1os8 (1.6 A, 90.1%, 0.86 A), 2fmj (1.6 A, 92.3%, 0.85 A), 1sgt (1.7 A, 88.7%, 0.88 A), 3i77 (2.1 A, 96.1%, 0.90 A),
2ahd	3.0	3.0	X				2	1s3l (2.4 A, 100.0%, 0.85 A), 1s3n (2.5 A, 100.0%, 0.36 A),
1jod	3.2	3.0	X		X		2	1f35 (2.3 A, 100.0%, 0.77 A), 1job (2.4 A, 96.9%, 0.89 A),
3i5s	3.0	3.0	X				2	3i5r (1.7 A, 100.0%, 0.80 A), 1pht (2.0 A, 100.0%, 0.55 A),
3fdf	3.2	3.2	X		X		2	3p9y (2.1 A, 93.2%, 0.79 A), 3omx (2.3 A, 94.2%, 0.85 A),
3fmv	3.3	3.3	X				2	3p9y (2.1 A, 93.2%, 0.62 A), 3omx (2.3 A, 94.2%, 0.82 A),

3n5u	3.2	3.2	X		X		6	3e7a (1.6 A, 100.0%, 0.51 A), 3egg (1.9 A, 100.0%, 0.52 A), 1jk7 (1.9 A, 98.0%, 0.70 A), 1it6 (2.0 A, 98.0%, 0.53 A), 2o8g (2.5 A, 97.6%, 0.48 A), 1s70 (2.7 A, 92.9%, 0.60 A),
2v9z	3.0	3.0	X				3	3g9x (0.9 A, 96.9%, 0.66 A), 3fbw (1.2 A, 97.3%, 0.77 A), 1bn7 (1.5 A, 96.9%, 0.72 A),
4ddf	3.1	3.1	X	X	X		3	3n79 (1.5 A, 95.1%, 0.59 A), 3pac (1.9 A, 88.0%, 0.75 A), 3vcd (2.4 A, 100.0%, 0.41 A),
3b1k	3.3	3.3	X			X	2	3b1j (2.2 A, 100.0%, 0.67 A), 3b20 (2.4 A, 100.0%, 0.86 A),
2ayg	3.1	3.1	X				2	1r8h (1.9 A, 100.0%, 0.73 A), 2aye (2.3 A, 100.0%, 0.67 A),
2ayb	3.2	3.2	X			X	2	1r8h (1.9 A, 100.0%, 0.69 A), 2aye (2.3 A, 100.0%, 0.69 A),
3red	3.0	3.0	X	X	X		2	1ju2 (1.5 A, 86.7%, 0.50 A), 3gdp (1.6 A, 86.7%, 0.44 A),
3g1k	3.1	3.1	X				9	3g91 (1.2 A, 99.6%, 0.54 A), 3g00 (1.7 A, 99.6%, 0.55 A), 3ga6 (1.9 A, 99.6%, 0.55 A), 3fzi (1.9 A, 100.0%, 0.58 A), 3g2d (2.3 A, 99.6%, 0.53 A), 3g2c (2.3 A, 100.0%, 0.61 A), 3g8v (2.4 A, 99.6%, 0.55 A), 3g0r (2.4 A, 99.6%, 0.55 A), 3g4t (2.6 A, 100.0%, 0.53 A),
1wac	3.0	2.9	X			X	2	1uvj (1.9 A, 99.7%, 0.52 A), 1hi8 (2.5 A, 95.9%, 0.60 A),
2g3k	3.0	3.0	X				2	2j9v (2.0 A, 98.9%, 0.50 A), 2j9u (2.0 A, 98.9%, 0.40 A),
4dfs	3.8	3.8	X		X		3	3azy (1.6 A, 99.2%, 0.55 A), 3azx (1.8 A, 99.2%, 0.48 A), 3azz (1.8 A, 99.2%, 0.54 A),
2bvg	3.2	3.2	X		X		2	2bvf (1.9 A, 100.0%, 0.52 A), 2bvh (2.9 A, 100.0%, 0.48 A),
2abm	3.2	3.2	X				2	2o9g (1.9 A, 98.7%, 0.43 A), 2o9d (2.3 A, 98.7%, 0.42 A),
